# Oesophageal cancer multi-disciplinary tool: a co-designed, externally validated, machine learning tool for oesophageal cancer decision making

**DOI:** 10.1016/j.eclinm.2025.103527

**Published:** 2025-09-30

**Authors:** Navamayooran Thavanesan, Mohammad Naiseh, Miguel Terol, Saqib Andrew Rahman, Samuel Luke Hill, Charlotte Parfitt, Zoë S. Walters, Sarvapali Ramchurn, Sheraz Markar, Richard Owen, Nick Maynard, Tayyaba Azim, Zehor Belkatir, Elvira Vallejos Perez, Mimi McCord, Tim Underwood, Ganesh Vigneswaran

**Affiliations:** a Innovation for Translation Research Group (ITRG), School of Cancer Sciences, Faculty of Medicine, University of Southampton, United Kingdom; b Department of Computing and Informatics, Bournemouth University, United Kingdom; c University Hospitals Southampton NHS Foundation Trust, United Kingdom; d School of Electronics and Computer Science, University of Southampton, United Kingdom; e Nuffield Department of Surgical Sciences, University of Oxford, United Kingdom; f Department of Oesophagogastric Surgery, Churchill Hospital, Oxford University Hospitals NHS Trust, United Kingdom; g School of Computer Science, Horizon Digital Economy Research, University of Nottingham, United Kingdom; h Heartburn Cancer UK, United Kingdom

**Keywords:** Oesophageal cancer, Multidisciplinary teams, MDT, Machine learning, Artificial intelligence, Decision support tool

## Abstract

**Background:**

The oesophageal cancer (OC) multi-disciplinary team (MDT) operates under significant pressures, handling complex decision-making. Machine learning (ML) can learn complex decision-making paradigms to improve efficiency, consistency, and cost if trained and deployed responsibly. We present an externally validated ML-based clinical decision support system (CDSS) designed to predict OC MDT treatment decisions and prognosticate palliative scenarios, co-designed using Responsible Research and Innovation (RRI) principles.

**Methods:**

Clinicopathological data collected from 1931 patients between 4th September 2009, and 8th November 2022 were used to test and validate models trained through four ML algorithms to predict curative and palliative treatment pathways along with palliative prognosis. 953 OC cases treated at University Hospitals Southampton (UHS) were used to train ML models which were externally validated on 978 OC cases from Oxford University Hospitals (OUH). Model performance was evaluated using Area Under Curve (AUC) for treatment classifiers and calibration curves for survival models. A parallel RRI program at the University of Southampton (United Kingdom) combining clinician interviews and inter-disciplinary workshops was conducted between 16.3.23 and 23.5.24. The RRI program comprised a group of 17 domain experts comprising programmers, computer scientists, clinicians and patient representatives to allow end-users to contribute towards the co-design of the CDSS user interface.

**Findings:**

Cohorts differed in baseline characteristics, with the external cohort (OUH) being younger, having better performance status, and a higher prevalence of pulmonary and vascular disease. Despite these differences, on internal validation (UHS cohort) mean AUCs for the primary treatment model were: MLR 0.905 ± 0.048, XGB 0.909 ± 0.044 and RF 0.883 ± 0.059 (k = 5 cross-validation) and MLR 0.866 (95% CI 0.866–0.867), XGB 0.863 (0.862–0.864), RF 0.863 (0.867–0.868) on bootstrapped resampling. For the palliative classifier, mean AUCs were: MLR 0.805 ± 0.096, XGB 0.815 ± 0.081 and RF 0.793 ± 0.083 (k = 5 cross-validation) and MLR 0.736 (95% CI 0.734–0.737), XGB 0.799 (0.798–0.800), RF 0.781 (0.778–0.782) on bootstrapped resampling. On external validation (OUH cohort), AUCs were MLR 0.894, XGB 0.887 and RF 0.891 for the primary treatment model and MLR 0.711, XGB 0.742 and RF 0.730 for the palliative treatment classifier. Predicted survival probability from the palliative survival model was well calibrated over the first 12 months post-diagnosis in both cohorts. The RRI program provided a collaborative environment leading to valuable modifications to the CDSS including prediction explanations, visual aids for survival and integrated education for users producing a user-friendly and quick to use tool.

**Interpretation:**

We present a novel, responsibly developed, externally validated AI CDSS trained to predict oesophageal cancer MDT decisions. It represents the foundations of a transformative application of ML, personalised, consistent and efficient MDT decision-support within OC which aligns to RRI principles.

**Funding:**

Doctoral Studentship for NT (Institute for Life Sciences (University of Southampton) & University Hospital Southampton), 10.13039/100014013UKRI TAS Pump-Priming Grant (TAS_PP_00167).

**Research in context**

**Evidence before this study**

Machine learning (ML) a branch of Artificial Intelligence (AI) may offers a viable solution towards supporting clinicians however to date no externally validated models have been reported within Oesophageal cancer (OC). We searched PubMed on August 27th, 2025, without date or language restrictions for publications using the terms “Machine Learning” AND “Oesophageal cancer” AND “Multidisciplinary Team” (or “Cancer Board” or “Tumour Board”). We did not identify any additional studies beyond those previously published by this research group investigating ML as a means of predicting treatment assignment at MDT for OC.

**Added value of this study**

The machine learning algorithms used within this study are easily accessible, off-the-shelf libraries and compatible within the current digital healthcare infrastructures of many countries worldwide. The resulting CDSS, which provides both treatment classification and palliative prognostication has been externally validated using data from a separate geographical catchment. Finally, the parallel Responsible Research and Innovation (RRI) program, has integrated early input from stakeholders in the development process.

**Implications of all the available evidence**

Our results suggest that ML can learn and predict MDT treatment decisions effectively in OC posing significant implications for future-proofing MDT operations against continued rises in caseload both within OC as well as other cancer types. Future iterations can also adapt to novel molecular markers and treatment modalities. The CDSS here provides rapid decision support for OC MDT personnel as well as a platform with which to counsel patients.

## Introduction

Oesophageal cancer (OC) is the 7th commonest cause of cancer death worldwide and is a cancer of unmet need.^1^^,^^2^ Affected patients commonly present beyond their late 60s, are nutritionally compromised and often co-morbid. They require high-quality decision-making as treatment options have grown in number and complexity, each carrying significant survival and quality of life implications.^3^ Cancer multidisciplinary teams (MDTs), while greatly improving patient outcomes, face a relentless increase in caseload and clinical complexity.^4^ They are susceptible to pressured, inconsistent and potentially suboptimal decision-making.^5^^,^^6^

In 2017, Cancer Research UK evaluated UK MDT services finding an urgent need for evolution and adaptation within their operational framework.^4^ Their report stressed an aging population combined with expanding treatment options had led to caseload volumes rising linearly with almost no corresponding increase in MDT resources to adapt or cope, a scenario common to many economies and countries. MDTs had on average 2–3 min to discuss cases, with no additional time to audit, reflect or learn from their internal decision-making. The MDT's challenges are also financial: the national cost of MDTs in the United Kingdom was estimated at £50 million in 2010, £88 million in 2011/12, approximately £150 million by 2014/2015 and £316 million as of 2024.^4^^,^^7^^,^^8^ While this data is now over a decade out of date, there is nothing to indicate that the situation has improved in that time with regards to cost or case discussion time. Furthermore, assuming a starting NHS consultant salary of approximately £100,000 p.a., a 3-h MDT would cost at minimum £7500 per consultant present per year (with a minimum of 4–5 consultants present being typical of most MDTs). Reducing an MDT by even an hour could provide a hospital significant savings over a calendar year.

A process to streamline, prioritize, and ease MDT caseload is essential within the current economic climate of many world regions. Artificial intelligence (AI) has seen a boom in healthcare use-cases in the form of clinical decision-support systems (CDSS).^9^, ^10^, ^11^, ^12^ Machine learning (ML), a branch of AI which leverages advanced computational power to identify patterns within complex and multimodal data has provided one such engine for CDSSs and its potential to support OC management has been recently recognized.^13^^,^^14^ ML has seen increasing adoption within early detection of cancer^15^, ^16^, ^17^ yet while AI platforms have been applied to MDT-style frameworks in some medical fields, OC MDTs have remained untouched in this regard.^11^, ^12^, ^13^^,^^18^ Similarly, a paucity of qualitative evidence exists on the viewpoints of clinicians and patients on the use of AI CDSSs in OC which creates a knowledge gap when design such tools for translation. Medical AI (MAI) necessitates trustworthy, ethical and responsible innovation.^19^ Where much of the literature has focused on proving MAI tools, there is a paucity of consideration for their implications on stakeholders from design-to-deployment.^19^ These include governance, handling bias, quality control, data drift detection and AI explainability.^20^ Responsible Research and Innovation (RRI) has developed in recent years to address this, aiming to maximise societal benefit while minimizing harm.^21^ The AREA framework (Anticipation, Reflection, Engagement and Action) is an example of this which integrates RRI within the life cycle of research programs.^21^

Within this study we present a novel, responsibly developed, externally validated AI CDSS trained to predict oesophageal cancer MDT decisions. The tool utilizes readily accessible, off-the-shelf ML algorithms built into a user-friendly interface. The CDSS was co-designed with Patient & Public Involvement (PPI), clinicians, and computer scientists specialising in AI. By harnessing AI-based technologies in a bid to replicate and simulate OC MDT decision-making ML may be able to offer the potential to streamline, standardize and increase efficiency within the OC MDT operational framework in a manner which still aligns with Responsible AI (RAI) principles.

## Methods

This was a mixed-methods study including a retrospective complete-case analysis of oesophageal cancer patients across two tertiary referral centres in the UK (University Hospital Southampton and Oxford University Hospitals) under the ethical approvals of IRAS 233065 & 319540.

### Study cohort

#### Training cohort

Oesophageal cancer patients discussed at MDT at University Hospital Southampton (UHS) between 2010 and 2023 were identified from a prospectively maintained local database and unit submission records to the UK National Oesophagogastric Audit (NOGCA). Treatment decisions were based on UK National Institute for Clinical Excellence (NICE) guidelines.^13^^,^^22^ Patients who present with non-metastatic disease (T0-4, N0-3, M0 disease) and fit (determined by the referring clinician and ratified by the MDT) for neoadjuvant therapies and/or surgery are filtered down curative pathways. For those with metastatic disease at presentation, or who are non-metastatic but felt too unfit for curative treatment are managed with palliative intent which may also be filtered based on their performance status (PS 0–2 patients for example, are deemed eligible for 1st-line palliative chemotherapy by NICE).

The mainstay of curative treatment for locally advanced OC is surgical resection alone (designated “Surgery”) or surgery combined with neoadjuvant therapy (NAT) (neoadjuvant chemotherapy (designated “Chemo”) or neoadjuvant chemoradiotherapy (designated “CRT”)). While a small proportion of patients detected early are eligible for endoscopic resection, their management remains controversial and entry to the MDT, nuanced meaning they could not be standardized to allow a fair comparison.^23^ While they were excluded from the external validation process, the results of a UHS model incorporating endoscopic resection are presented separately within the supplementary materials. Definitive CRT as monotherapy was also excluded from this study owing to insufficient training data for meaningful modelling.

In general, non-curative patients are offered one of five possible outcomes: best supportive care (designated “BSC”), palliative chemotherapy (designated “Chemo” within the palliative models), palliative radiotherapy (designated “RTX”, typically to either the primary tumour and/or symptomatic secondary sites amenable to radiotherapy, however for the purposes of this study, RTX was defined as therapy to the primary tumour), palliative oesophageal stent alone, or with an oncological adjunct (chemotherapy or radiotherapy, and designated “Stent_Onc”).

Predictor variables for model training were derived from clinicopathological variables known to be routinely considered by the MDT. Clinical staging was assessed on baseline imaging (Computer Tomography (CT) and/or Positron Emission Tomography (PET)) and tissue biopsies in accordance with the American Joint Committee on Cancer (AJCC) Tumour-Node-Metastasis (TNM) staging system (7th edition until 2017 and 8th edition thereafter). Novel molecular markers and immunotherapies which have been approved for metastatic disease in the UK since 2021 were not built into this first generation of models as these are emerging treatments and consequently there was insufficient training data for inclusion.

#### External validation cohort

The validation cohort were identified from a prospectively maintained clinical database (Cancer Outcomes Database Application for Upper GI or “CODA-UGI”) at Oxford University Hospitals (OUH) which was similarly submitted to NOGCA. The included patients were discussed at MDT over the same study period and underwent the same inclusion/exclusion criteria as the training cohort.

### Ethics

This research (including all relevant participant informed consents) was conducted under the following ethical approvals; The United Kingdom Heath Research Authority (HRA) Integrated Research Application Systems (IRAS) 233,065 & 319,540 as well as under the approval of the local ethical review board: University of Southampton Ethics Research & Governance Online (ERGO) 70,735. Anonymised external validation data access was granted after review by CODA-UGI data access committee, and following registration and approval via the Oxford University Hospitals governance platform (project no. 8441).

### Statistics

#### Patient sample

Sample size was dictated by the number of retrospectively recorded cases available for analysis at both centres. As a specific “effect” is not sought here from comparing treatment outcomes, a sample size calculation was not relevant to this use case. We set a historical boundary at 2010 to ensure we balanced maximising sample size while ensuring treatment paradigms remained relevant and still in-practice within the modern era.

#### Cohort comparison

Differences between the training and validation cohorts were assessed using Standardised Mean Difference (SMD). An SMD of 0.2 was deemed a small difference, 0.5 a medium difference and 0.8 a large difference.

Numeric performance metrics where relevant are presented as mean ± standard deviation (SD) and mean ± standard error from the mean (SEM) for thr 5-fold cross-validated models. Where model performance has been tested with bootstrapped resampling, 95% confidence intervals have also been provided.

#### Model comparison

Differences in performance between algorithms were analysed using the Kruskal–Wallis test coupled with the Pairwise Wilcoxon Rank Sum Test where appropriate (p values were adjusted using the Benjamini-Hochberg correction, (p < 0.05 was deemed significant)).

### Machine learning model development

#### Data preparation and analysis

Data analysis, model training and validation were conducted in R (version 4.2.2) with relevant packages described where first used (Supplemental Materials). The features used in this study (Table 1) are derived from a combination of domain expertise and UK national guidelines.^22^ Data was manually checked for quality control by NT and CP. Data entry was standardised for analysis using terminology accepted within the clinical field. As this was a complete analysis, any missing data was retrospectively extracted from hospital electronic health records to ensure high-fidelity quality control. Age and overall survival were treated as continuous variables, while the remaining covariates were categorical (Table 1). Three separate decision-assistance models were developed: a primary classification model which triaged patients into either a specific curative pathway directly or triaged to a secondary, bespoke, palliative treatment classification model. A third, survival model was also trained to predict prognosis for a palliative patient from time of diagnosis when factoring in palliative treatment. Survival analysis was first undertaken using a Kaplan–Meier survival estimator (“survival” package). Median survival was stratified by treatment with a log-rank test-of-significance between curves. Overall survival was defined as survival from date of diagnosis to date of death or last recorded follow-up.

**Table 1:** Demographics for the training cohort (UHS) and validation cohort (OUH).

Pre-treatment variables	UHS (N = 953) (%)	OUH (N = 978) (%)	Test SMD
**Gender**			
Male	718 (75.3%)	744 (76.1%)	0.017
Female	235 (24.7%)	234 (23.9%)	
**Median age in years (Range)**	70.0 (21.0–96.7)	68 (29.0–96.0)	0.156
**Performance status**			
0	371 (38.9%)	712 (72.8%)	0.726
1	329 (34.5%)	150 (15.3%)	
2	160 (16.8%)	71 (7.3%)	
3	88 (9.2%)	43 (4.4%)	
4	5 (0.5%)	2 (0.2%)	
**cT stage**			
0	4 (0.4%)	0	0.885
Is	3 (0.3%)	0	
1 (unspecified)	7 (0.7%)	2 (0.2%)	
1a	1 (0.1%)	13 (1.3%)	
1b	1 (0.1%)	17 (1.7%)	
2	169 (17.7%)	196 (20.0%)	
3	557 (58.4%)	503 (51.4%)	
4 (unspecified)	134 (14.1%)	7 (0.7%)	
4a	37 (3.9%)	138 (14.1%)	
4b	15 (1.6%)	72 (7.4%)	
X	25 (2.6%)	30 (3.1%)	
**cN stage**			
0	254 (26.7%)	313 (32.0%)	0.340
1	437 (45.9%)	310 (31.7%)	
2	183 (19.2%)	253 (25.9%)	
3	61 (6.4%)	97 (9.9%)	
X	18 (1.9%)	5 (0.5%)	
**cM stage**			
0	690 (72.4%)	712 (72.8%)	0.047
1	257 (27.0%)	263 (26.9%)	
X	6 (0.6%)	3 (0.3%)	
**Tumour location**			
Proximal Oesophagus	22 (2.3%)	20 (2.0%)	0.885
Mid oesophagus	102 (10.7%)	176 (18.0%)	
Distal Oesophagus	570 (59.8%)	321 (32.8%)	
Siewert 1	56 (5.9%)	256 (26.2%)	
Siewert 2	124 (13.0%)	205 (21.0%)	
Siewert 3	57 (6.0%)	0	
Siewert undefined	22 (2.3%)	0	
**Tissue histology**			
Adenocarcinoma	749 (78.6%)	780 (79.8%)	0.029
Squamous Cell	204 (21.4%)	198 (20.2%)	
**Co-morbidities**			
Chronic pulmonary disease (CPD)	130 (13.6%)	179 (18.3%)	0.128
Peripheral vascular disease (PVD)	43 (4.5%)	23 (2.4%)	0.119
Cerebrovascular disease (CVD)	106 (11.1%)	44 (4.5%)	0.249
Uncomplicated diabetes (DM uncomp)	128 (13.4%)	155 (15.8%)	0.068
Leukaemia	4 (0.4%)	1 (0.1%)	0.062
Lymphoma	11 (1.2%)	13 (1.3%)	0.016
Renal disease	39 (4.1%)	34 (3.5%)	0.032

Standardized Mean Differences (SMD) are provided for the two cohorts. An SMD of 0.2 is considered a small difference, 0.5 medium and 0.8 or more, a large difference.

#### Feature selection

The features used in this study are derived from a combination of domain expertise and UK national guidelines.^22^ The features outlined in Table 1 are common to both the full cohort model and the palliative models except for the additional “obstructing” variable within the latter which was defined as either severe dysphagia to solids and liquids or difficulty passing the gastroscope at the time of the original diagnostic gastroscopy (while dysphagia of some degree is a hallmark of OC even in curative settings, cases which are deemed curative at diagnosis have rarely progressed to a stage where the lesion is causing severe dysphagia or an inability to pass a gastroscope which is more typically of palliative cases). The final palliative treatment allocation was then included as an extra feature within the palliative survival models. Feature selection was primarily dictated by the clinical variables routinely collected at the respective training and validation units (this was to ensure a pragmatic access to realistically accessible variables combined with domain knowledge of variables routinely discussed at MDT. Race, BMI, smoking status for instance are not routinely discussed or considered beyond exception circumstance (in situations of extremely high BMI which may make surgery more challenging or risky for instance). Similarly, while the American Society of Anaesthesiology (ASA) grading system is assessed pre-operatively in all surgical candidates, this score is not used in those not undergoing surgery or those who are palliative. As such their performance status is a more practical variable as it is considered across treatment pathways.

#### Machine learning algorithms

The ML algorithms used in this study were chosen for several reasons: firstly, they allowed us to focus explainable, accessible and technically realistic ML architectures which can be implemented easily within current healthcare systems. In many world regions (including the UK) these systems are under immense financial and technological restrictions. Deep learning platforms were avoided as they are too opaque for this level of high-stakes decision-making, and too complex for easy implementation while still allowing regulators and hospital clinicians ready access to the explainability of the final decisions. Furthermore, high quality, clean, clinical data is notoriously difficult to curate at the scales needed for deep learning platforms which typically demand thousands if not tens of thousands of data points for quality learning, making standard architectures which can handle smaller datasets instantly more favourable. Finally, it is established that within tabular data structures, ML algorithms such as tree-based models outperform deep learning architectures when provided tabular data.^24^ Multinomial Logistic Regression, Random Forests and eXtreme Gradient Boost models were trained through “caret” package using “nnet”, “RandomForest”, and “xgboost” libraries respectively.^25^ Survival modelling used Random Survival Forests as these have been shown to outperform traditional Cox Proportional Hazard models for prognostication in OC patients post-oesophagectomy (randomForestsSRC package).^14^^,^^26^

#### Model training

Classifier models were trained in the “caret” package in R using the train () (the “method = ” argument was determined by the base algorithm, “metric” was set to “logloss” and the “trControl” argument applied). The trainControl () function was used with “method = cv”. A 5× cross validation was set with the train and test folds from each indexed for tracking of predictions. The test fold predictions were then saved and averaged to provide individual ROC curves for each outcome class with 1× standard error of the mean (the rationale for this is described in the next section). A manual ROC for each class was generated over a single Multinomial ROC as this provided insight into which classes were best or least confidently discriminated. Additionally, internal metrics on balanced accuracy were obtained using the resamples () function (“caret” package) and averaged across the 5-fold CV models.

The palliative survival model was trained using the rfsrc () training function (“randomForestSRC” package, ntree = 1000, “nodesize=” was set based on the tune () function (ntreetry = 200)).

Model hyperparameters for all final models will be provided within the Supplementary Results.

#### Validation and model performance

Internal validation for the treatment classifier models was by k = 5-fold cross-validation (“caret” package) to provide estimated generalizability error averaged across test sets in each fold. The final model for each algorithm was then trained on the full training cohort and tested on the full OUH validation cohort (external validation). Classifier models were optimised for log loss during training and their mean-model performance assessed primarily on balanced accuracy (accuracy weighted by class size) and area under the curve (AUC of the Receiver Operator characteristic (ROC)) for each outcome class (one versus rest) using default probability thresholds set by the caret package. As 5-fold cross validation was used (to optimise a balance between sufficient diversity in the test folds without reducing training set sample size unduly) providing 5 sample metrics, 95% confidence intervals are not provided here as they assume a normal distribution (c.f. Kwak et al., 2017^2^) and the law of large numbers and central limit theorem typically requires at least 30 samples for this to be testable. Importantly, the need for estimating generalisability error within the training set is largely obviated by a truly independent external validation set (oxford cohort) providing a direct assessment of generalisability. A standard error of the mean however is provided across these thresholds on the visual ROC plots for error estimation. To statistically test for differences in performance between classifier algorithms, AUCs were also generated over 1000 bootstraps (models were trained on the bootstrapped sample and tested on the out-of-bag cases). Mean, standard deviation, range and 95% confidence intervals are provided for the bootstrapped model AUCs. Differences in performance between algorithms were analysed using the Kruskal–Wallis test coupled with the Pairwise Wilcoxon Rank Sum Test where appropriate (p values were adjusted using the Benjamini-Hochberg correction, (p < 0.05 was deemed significant)).

Survival forests were internally validated using bootstrapped resampling (1000 forests, ntree = 1000 per forest) with hyperparameter tuning via the Tune () function. Mean-model performance was assessed primarily on calibration, while additional metrics: Prediction error and Continuous Rank Probability Score (CRPS) are also provided.

Calibration curves were plotted both by quintile (based on survival probability at a single time point), and by event-probability at 3,6, and 12 months (“pec” package). Quintile-based survival curves were derived from mean test-set predictions averaged at each time point across all bootstrapped models and plotted against the corresponding Kaplan Meier (observed) survival probability. Cases were stratified into quintiles based on predicted 1-year survival using the RSF model with Q1 being highest risk (0–20% predicted survival) versus Q5 being lowest risk of death at 1-year (80–100% predicted survival). The predicted survival over 5 years is then plotted for each subgroup (the x-axis) as 5-year survival is a standard survival metric within oncology. This approach is again based on Rahman et al.^3^ Quintile-based plots provide evaluation of the model when patients are stratified by risk at a single defined time-point, while calibration plotted at sequential time-points allow for comparison of predictions across the cohort at multiple timepoints. This combined approach offers clearer insight into the optimal operating window for the model longitudinally and by patient-risk.

Prediction error was defined as 1- Concordance.^4^ Here, concordance is the percentage of observation-pairs where the probability of a true event is greater than a true non-event (a perfect model error rate = 0).^5^ Error rate was extracted for each bootstrapped model and averaged.

The Continuous Rank Probability Score (CRPS), (defined as Integrated Brier Score divided by time) is another measure of prediction calibration and derived from the Brier score (mean squared difference between predicted probability and observed probability^6^). In this study it was averaged across all bootstrapped models.^4^ A perfect model scores 0 and a perfectly inaccurate model scores 1.

Model fairness was not a primary outcome in this study however the impact of age on OC treatment allocation has been previously investigated.^27^

### Responsible co-design

To ensure the applicability and real-world utility of the CDSS we pursued an RRI program in parallel to the CDSS development. Heartburn Cancer UK, a leading charity for oesophageal cancer provided PPI, offering insight into the patient experience. Our approach involved early engagement with clinicians and computer scientists to ensure the tool was clinically relevant, technically sound and user-friendly. Regular RRI workshops were combined with semi-structured interviews using MDT domain experts. These are detailed within the Co-Design section of the Supplementary Methods.

### User Interface

Using insights from our RRI program, we developed a high-fidelity prototype of the User interface (UI) using the “Shiny” R package. Trained models were uploaded with their performance metrics, Receiver Operator Characteristic (ROC) curves and a short educational summary of the performance metrics. The Palliative Survival model is presented using treatment-specific survival curves for the recommended palliative pathway and a user-selectable alternative pathway to provide a visual comparison of the potential prognoses. For classifier models, a Local Interpretable Model-Agnostic Explanation (LIME, “LIME package”) was integrated to provide prediction explanations in real-time. LIME was used within the prototype UI as the package currently supports a diverse array of ML models through the “caret” package.

### Role of the funding source

The funding sources were not involved in study design, data collection, analysis, interpretation of data or writing of this manuscript.

## Results

### Cohort demographics

A total of 1047 eligible UHS cases were identified of which 94 were excluded for endoscopic resection, leaving 953 cases for training the initial model. Within the palliative sub-group (N = 439), two were excluded from the palliative-specific models as they were assigned a non-standard chemoradiotherapy regimen. As the initial model does not need to provide a specific palliative treatment however, they were eligible for inclusion within the primary model to maximise training data.

Within the validation cohort, a total of 978 eligible cases were identified and provided by OUH of which 475 palliative cases were identified for validation the palliative models. The Training Cohort (UHS) and the Validation cohort (OUH) are outlined in Table 1. Detailed demographic breakdown by outcome class is provided in Supplemental Table S1 while palliative cohort demographics are provided in Supplemental Table S2.

The two cohorts differed in composition across several variables including age, performance status, cT and cN stage, tumour location, and incidence of chronic pulmonary disease, peripheral vascular disease and cerebrovascular disease (Supplemental Table S3). In summary the OUH cohort presented a typically younger, physically more active cohort despite a higher incidence of pulmonary and vascular disease. The distribution of biological gender, cM staging at presentation, tumour histology and incidence of the remaining co-morbidities were consistent in both cohorts.

### CDSS model performance

#### Primary treatment model classification performance

**Fig. 1:**
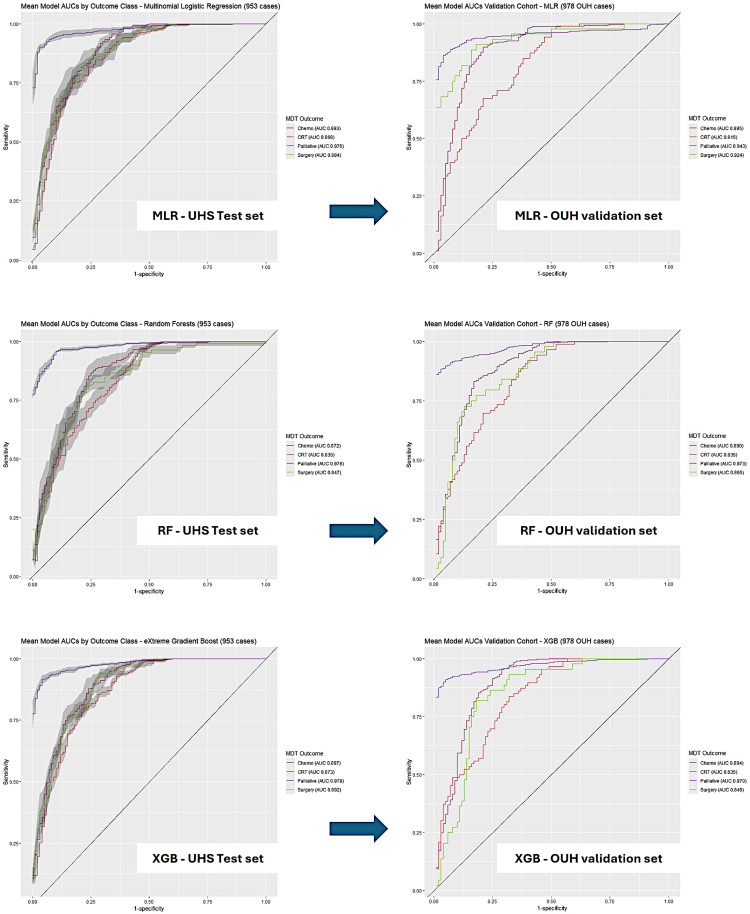
Mean cross-validated ROC curves for each classifier algorithm (UHS cohort on left, OUH validation cohort right). Shaded areas represent ±1× Standard error from the Mean.

Performance of the primary treatment classifiers were first tested internally on 5-fold cross validation and then externally on the OUH cohort to confirm generalisability. All three algorithms exhibited strong classification performance within the primary model. The XGB model performed best on internal cross-validation with a mean AUC of 0.909 ± 0.044 while the MLR model generalised best with a validation set performance of 0.894 ± 0.056. Mean balanced accuracy calculated for MLR, RF and XGB algorithms were 0.780 ± 0.008, 0.752 ± 0.019 and 0.781 ± 0.009 respectively (Table 2, Fig. 1). Over 1000 bootstraps, MLR and RF performed statistically better than XGB (Supplemental Tables S4 and S5) however the differences in mean performance remained modest. Performance was also assessed on an internally validated UHS model which incorporated endoscopic resection (Supplemental Figure S1, Supplemental Table S6).

**Table 2:** Mean classification performance AUCs for the UHS test set versus OUH validation set.

UHS model	UHS (N = 953) OUH (N = 978)	“Chemo”	“CRT”	“Surgery”	“Palliative”	Mean (±SD)
MLR	UHS test set	0.893	0.868	0.884	0.976	0.905 ± 0.048
	OUH validation set	**0**.**895**	0.815	**0**.**924**	0.943	**0**.**894 ± 0**.**056**
XGB	UHS test set	**0**.**897**	**0**.**873**	**0**.**892**	**0**.**979**	**0**.**909 ± 0**.**044**
	OUH validation set	0.894	**0**.**835**	0.849	0.970	0.887 ± 0.061
RF	UHS test set	0.872	0.835	0.847	0.978	0.883 ± 0.065
	OUH validation set	0.890	0.835	0.865	**0**.**973**	0.891 ± 0.059

Best performance for each class within the UHS training and OUH validation sets is highlighted in bold.

#### Palliative classifier performance

**Fig. 2:**
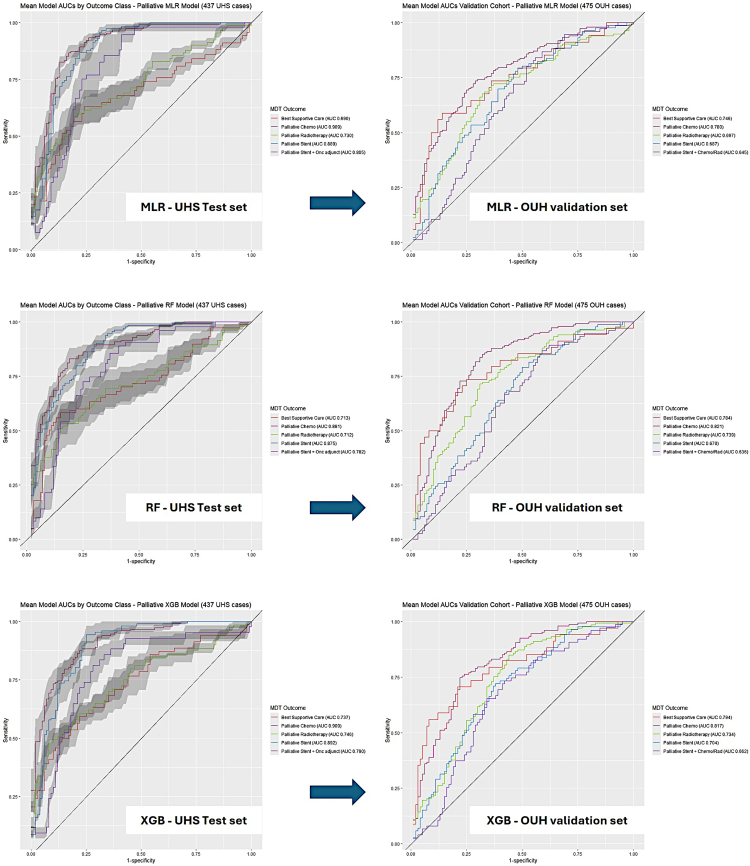
Mean cross-validated ROC curves for each palliative classifier algorithm (UHS cohort on left, OUH validation cohort right). Shaded areas represent ±1× Standard error from the Mean.

Palliative classifier performance was then assessed in a similar manner. All algorithms performed well in classifying palliative treatment (Table 3). The XGB algorithm offered the best performance on both the UHS and OUH datasets. Balanced accuracy for MLR, RF and XGB were 0.689 ± 0.018, 0.683 ± 0.028, 0.690 ± 0.013 respectively (Table 3, Fig. 2). Over 1000 bootstraps, XGB again performed statistically best (Supplemental Tables S7 and S8).

**Table 3:** Mean palliative treatment classification performance AUCs for the UHS test set versus OUH validation set.

UHS model	UHS (N = 437) OUH (N = 475)	“Chemo”	“BSC”	“RTX”	“Stent”	“Stent_Onc”	Mean
MLR	UHS	**0**.**909**	0.690	0.730	0.889	**0**.**805**	0.805 ± 0.096
	OUH Validation	0.780	0.746	0.697	0.687	0.645	0.711 ± 0.053
XGB	UHS	**0**.**909**	**0**.**737**	**0**.**746**	**0**.**892**	0.790	**0**.**815 ± 0**.**081**
	OUH Validation	0.817	**0**.**794**	0.734	**0**.**704**	**0**.**662**	**0**.**742 ± 0**.**064**
RF	UHS	0.881	0.713	0.712	0.875	0.782	0.793 ± 0.083
	OUH Validation	**0**.**821**	0.784	**0**.**739**	0.670	0.636	0.730 ± 0.077

Best performance for each class are in bold.

#### Palliative survival model performance

**Fig. 3:**
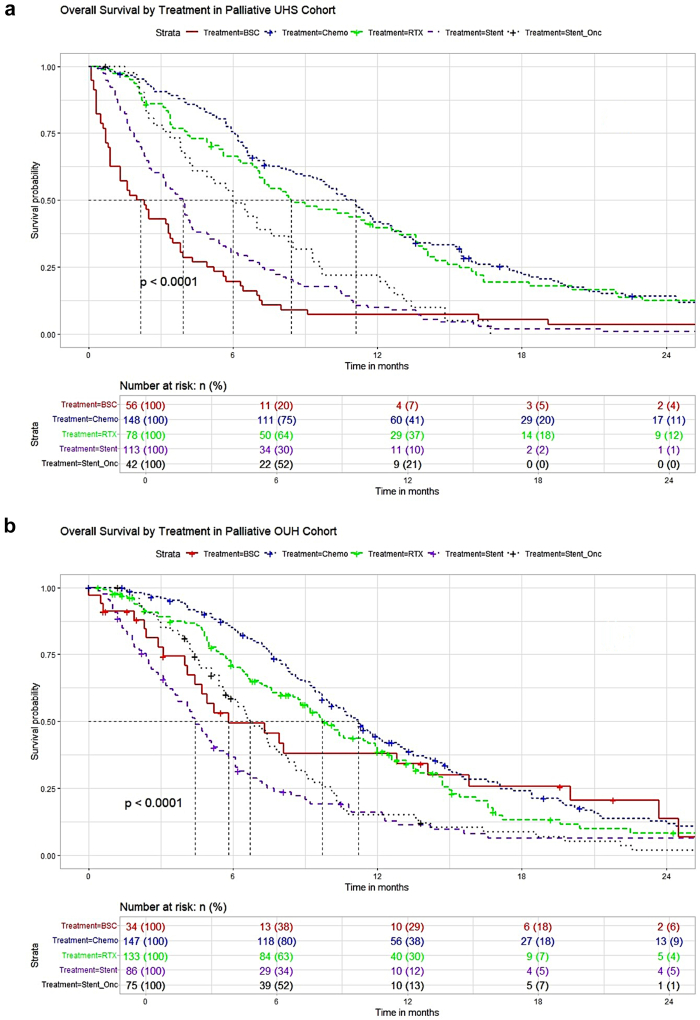
Kaplan Meier survival plots for the UHS cohort (a) and OUH cohort (b).

Palliative survival in both cohorts demonstrated significant survival differences between treatments (Fig. 3). Best median survival was associated with palliative chemotherapy in each cohort (UHS: median 11.1 months (95% CI 9.7–12.2), OUH 11.2 months (9.9–12.9)) followed by radiotherapy and stent ± oncological adjunct. However, while the stent only group survived longer in the UHS cohort, they experienced poorer outcomes relative to the BSC group within the OUH cohort. Supplemental Table S9 details median survival for both cohorts by treatment.

The final random survival forest model, trained on the full cohort after internal validation, demonstrated a prediction error of 0.331 and a CRPS of 0.077. On internal validation over 1000 bootstrapped models, mean prediction error was 0.334 ± 0.018 while mean CRPS was 0.112 ± 0.020. This was consistent with the validation cohort (Table 4).

**Table 4:** Survival model performance metrics for UHS and OUH cohorts with an interpretation of the performance metric provided.

Metric	Cohort	Score	Reference	Interpretation
Prediction error (1-Concordance)	UHS model	0.334 ± 0.017	0 = perfect concordance 1 = perfect non-concordance	Fair
	OUH validation set	0.354		Fair
CRPS (Integrated Brier Score/time)	UHS model	0.112 ± 0.020	0 = perfectly accurate model 1 = perfectly inaccurate model	Very Good
	OUH validation set	0.093		Very Good

**Fig. 4:**
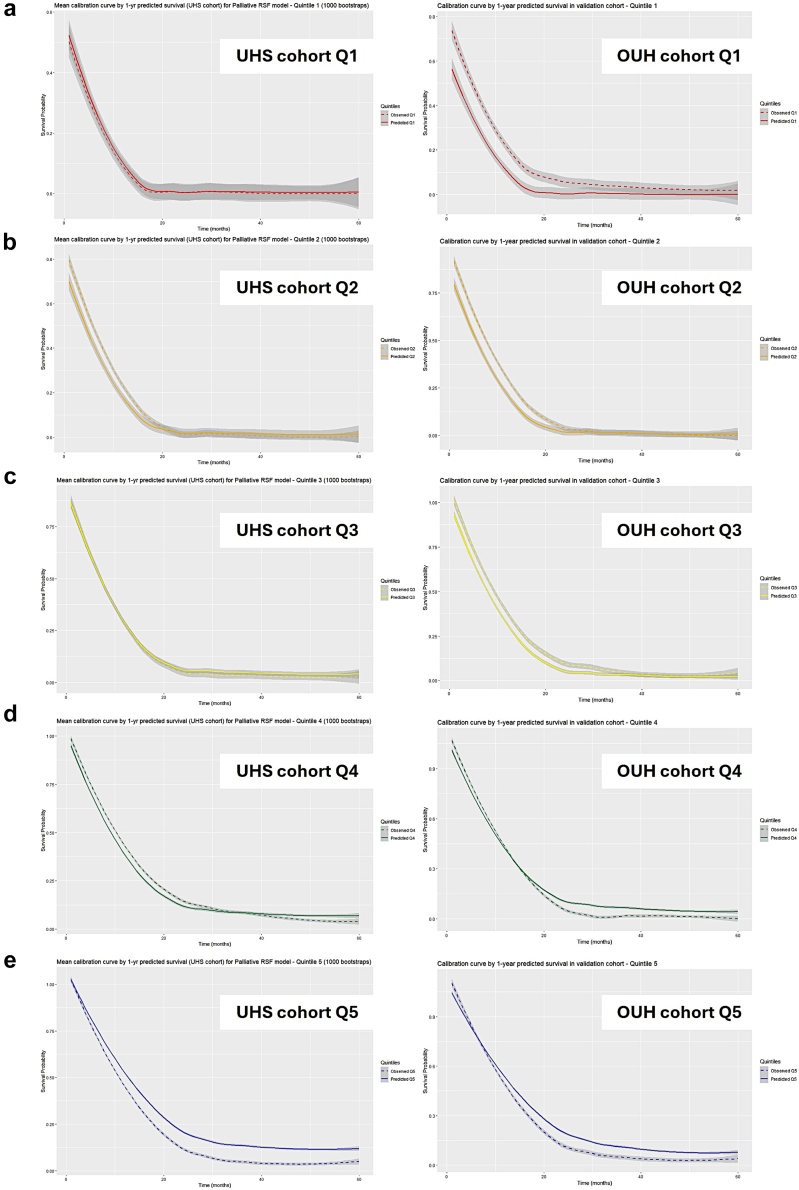
Quintile Calibration curves plotted with standard error over 60 months. Quintile cases are stratified based on predicted 1-year survival probability as determined by the RSF model (Quintile 1 = 0–20% (a), Quintile 2 = 20–40% (b), Quintile 3 = 40–60% (c), Quintile 4 = 60–80% (d), Quintile 5 = 80–100%).

**Fig. 5:**
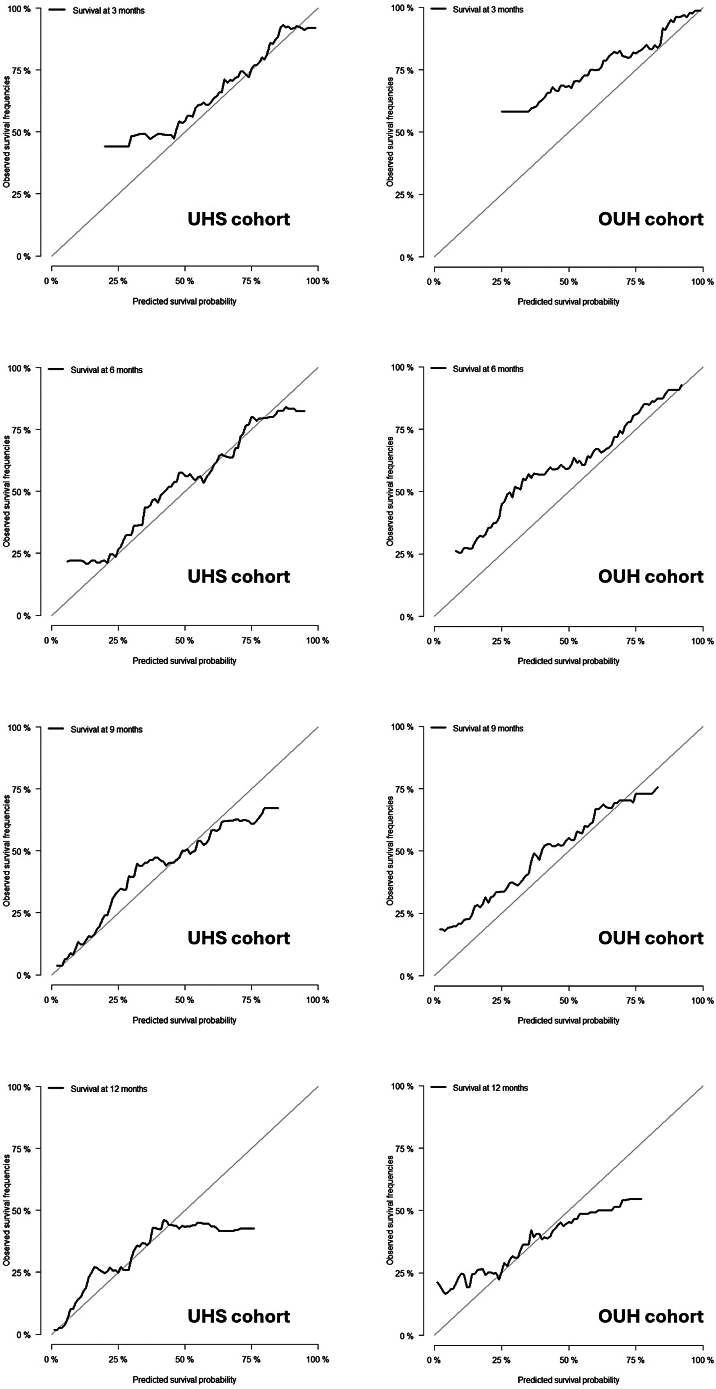
Calibration plots for the UHS cohort (left) and OUH validation cohort (right) at 3,6,9 and 12 months post-diagnosis.

Calibration curves were stratified by 1-year survival quintiles (Fig. 4) as well as by whole-cohort survival at sequential time points where calibration was best within the first 12 months (Fig. 5). Quintile-based analysis indicated calibration was best for the three highest-risk quintiles (Q1-3). Model predictions were pessimistic for Q4 patients but over-optimistic by Q5.

#### OUH models

To determine if the modelling process remained robust when applied to a non-UHS cohort the same algorithms were again trained using OUH as the training centre and tested on the UHS cohort as the external validation centre. XGB models again performed best on both internal and external validation across treatment classifiers (Supplemental Tables S10 and S11). Similarly, a survival model trained on the OUH cohort demonstrated good calibration within the first 12 months after which predictive performance dropped away (Supplemental Figure S3, Supplemental Table S12).

### Co-design insights

The co-design RRI program was set up to provide guiding insights into user-needs and concerns when implementing a clinical CDSS. This was stimulated by discussing prompts from the RRI card deck (Supplemental Figure S4) in combination with insights drawn from our clinician interviews (Interview questions provided in Supplemental materials) and RRI workshops. It highlighted several key challenges and considerations which were factored when developing the CDSS. Themes identified included: bias within the models, data drift, unintended inequalities of access, as well as safety and accuracy from a regulatory perspective. The RRI process recognised the impact CDSSs may have on clinical training for junior clinicians as MDTs are traditionally a source of experiential learning along with a need for education in AI literacy. Explainability proved a recurring theme along with the potential ramifications of group AI interactions where multiple human actors are interacting dynamically with the AI. This in turn prompted considerations over where the ultimate decision-making responsibility lies when a CDSS is supporting high-risk decision-making within healthcare. The themes identified through the RRI process are detailed in Tables 5 and 6 along with adjustments we gradually introduced into the tool to address these where possible.

**Table 5:** Thematic analysis of domain expert interviews highlighting user expectations, concerns and solutions engineered into the tool in response.

Clinicians' understanding of AI and its role in healthcare	Perceived potential benefits of AI in multidisciplinary teams (MDTs)	Barriers to the adoption and trust of AI in healthcare
**Theme 1: Conceptual Understanding and Knowledge Variability** The interviews revealed a significant variability in clinicians' understanding of artificial intelligence (AI) and machine learning (ML). Some clinicians demonstrated a deep understanding of these technologies, recognizing their potential and limitations, while others exhibited a more superficial or unclear perception. This disparity in understanding is likely to influence how different clinicians interact with and trust AI tools. As one clinician noted, **“Some of us see AI as just algorithms, but the deeper layers are often misunderstood.”** This variability suggests the need for targeted educational initiatives to ensure that all clinicians have a sufficient grasp of AI concepts.	**Theme 1: Improved Diagnostic Accuracy** Clinicians widely recognized AI's potential to improve diagnostic accuracy, particularly by analysing large datasets and identifying subtle patterns that may be overlooked by human clinicians. This capability was seen as a significant advantage, especially in the context of complex diseases like oesophageal cancer. **“AI can help us catch things we might otherwise miss in diagnostics,”** one clinician stated, highlighting the perceived value of AI in enhancing diagnostic precision. **Our Response:** We have ensured the tool's models have been internally and externally validated on a large training cohort	**Theme 1: Concerns About Bias and Accuracy** A significant barrier to the adoption of AI tools identified by clinicians was the concern about bias in AI algorithms and the accuracy of AI decisions. Clinicians expressed worry that AI could perpetuate or even exacerbate existing biases in healthcare, leading to unfair treatment decisions. One clinician emphasized, **“Bias in AI is a serious concern, especially if it leads to unfair treatment decisions,”** reflecting a common apprehension about the ethical implications of AI in healthcare. **Our Response:** We have identified and acknowledged the possibility of bias. The tool provides a detailed breakdown of the training cohort for user inspection. Future iterations will also include warning messages where specific clinical inputs represent cases with few data points e.g. “dementia = Y”
**Theme 2: AI as an Extension of Data Analytics** Several clinicians perceived AI as a powerful extension of traditional data analytics, capable of processing larger datasets and uncovering patterns that might escape conventional methods. This perception ties AI closely to evidence-based practice, where it is seen as enhancing the clinician's ability to make data-driven decisions. One clinician remarked, **“AI is like data analytics on steroids; it can handle much larger datasets and pick up on trends we might miss.”**	**Theme 2: Workload Reduction and Efficiency** Another major benefit identified was AI's potential to reduce clinicians' workload by automating routine tasks. This could allow clinicians to focus more on complex cases and patient interactions, thus improving overall efficiency in the healthcare setting. One clinician commented, **“AI could free us from repetitive tasks, giving us more time to focus on patients,”** emphasizing the role of AI in enhancing productivity. **Our Response:** We have integrated the ability to upload a list of patients simultaneously and provide a generate report of predictions for each patient in real time. This will allow use of the tool between MDT meetings too	**Theme 2: Transparency and Explainability** The need for transparency and explainability in AI decision-making processes was another critical barrier. Clinicians expressed a strong desire to understand how AI reaches its conclusions to trust and use these tools effectively. **“I need to know how AI makes its decisions before I can trust it,”** one clinician explained, underscoring the importance of AI interpretability in clinical practice. **Our Response:** The tool provides a detailed breakdown of the training cohort for user inspection; it also provides detailed performance metrics of the current models. We have integrated a LIME explanation plot that reactively updates in real time with user inputs to give a specific explanation at an instance level.
**Theme 3: Mystification and Misconceptions** The interviews also revealed that some clinicians hold misconceptions about AI, viewing it either as an almost omniscient entity or as an unreliable tool. This mystification of AI can lead to polarized views—some clinicians might place undue reliance on AI, while others might harbour unwarranted scepticism. As one participant explained, **“Some think AI is this magical tool that can do anything, while others don't trust it to do anything right.”** These misconceptions underscore the importance of clear communication about what AI can and cannot do in clinical settings. **Our Response:** We recognise that there remains an ongoing knowledge gap for Clinicians in the MAI sphere. While this is an evolving field, we have sought to assist the AI-lay clinician using the tool by providing a section which outlines some key metrics and a guide of their interpretation to allow them to critically appraise our model performance.	**Theme 3: Enhanced Decision Support** Clinicians also saw AI as a valuable tool for enhancing decision support, particularly in complex cases where multiple variables need to be considered. The ability of AI to process and analyse data rapidly was viewed as a way to formulate more comprehensive and informed treatment plans**. “AI could be a valuable assistant in making decisions in complicated cases,”** one clinician noted, underscoring the potential for AI to augment clinical decision-making.	**Theme 3: Impact on Clinical Autonomy** Concerns were also raised about the potential impact of AI on clinical autonomy. Clinicians worried that an over-reliance on AI might diminish the role of human judgment in decision-making, leading to a reduction in their autonomy. As one clinician put it, **“I'm worried that AI might take away our decision-making power, making us too dependent on it,”** reflecting a fear of losing control over clinical decisions. **Our Response:** We recognise this is a valid risk of automating a clinical decision-making framework like the MDT. Where the tool generates reports for a group of patients at once, it orders them in order of confidence in the recommendation. A traffic light system then signposts clinicians to cases of low confidence where the human is required to assess and recommend. This keeps the human central to the process for discussing those most difficult cases first and sense-checking high-confidence cases thereafter.
	**Theme 4: Personalized Medicine** The potential of AI to advance personalized medicine was another recognized benefit. Clinicians appreciated AI's ability to tailor treatments based on individual patient data, which could lead to better outcomes. **“With AI, we could move closer to truly personalized medicine, where treatments are tailored to the individual,”** one participant remarked, highlighting the transformative potential of AI in this area.	**Theme 4: Legal and Liability Concerns** Legal and liability concerns were also prominent among clinicians. They were unsure who would be held accountable if an AI tool made a mistake—whether the responsibility would fall on the clinician using the tool or the developer who created it. **“Who's responsible if AI makes a mistake? This is a big question to ask,”** one clinician stated, highlighting the legal uncertainties surrounding AI adoption. **Our Response:** We acknowledged the implications from a ethicolegal perspective and have included a disclaimer message at first use which explains clearly that responsibility of the decision remains with the human as it is a decision-support tool. This will remain the case even if certification as a medical device is
	**Theme 5: Predictive Analytics for Preventive Care** Clinicians acknowledged the potential of AI in predictive analytics, particularly for preventive care. AI could be used to identify patients at risk of certain conditions, enabling early intervention and improving patient outcomes. One clinician noted**, “AI could help us predict and prevent diseases by identifying at-risk patients earlier,”** indicating the proactive role AI could play in healthcare.	**Theme 5: Fear of Losing Skills** Finally, some clinicians expressed a fear that the adoption of AI could lead to a loss of skills, particularly in routine diagnostic tasks. There was concern that AI might replace certain aspects of their work, leading to skill degradation, especially among less experienced clinicians. **“There's a fear that AI could replace us in certain tasks, which may make some juniors lose some important skills,”** one participant observed, pointing to a potential unintended consequence of AI integration.

**Table 6:** Thematic analysis of RRI workshop outlining additional themes extracted on user expectations, concerns along with solutions engineered into the tool in response where relevant.

Themes	Computer scientists	Patient and public involvement
	**Theme 1: Explainable AI (XAI)** A major focus of the discussion has been on the tool more interpretable, leading to developments in explainable AI (XAI). This ensures that models can be understood and trusted by non-experts. *Supporting Code:* “*We've built tools that provide explanations for AI decisions, making it easier for users to trust the system.*”	**Theme 1: Ethical concerns** This includes issues such as data privacy, and fears of AI exacerbating inequality, might act as barriers to public acceptance. Supporting Code: “Who controls the data when AI is involved? This is my biggest concern.” **Our response:** By working symbiotically with the local hospital who provides the clinical data we also ensure it is stringently protected, anonymised as soon as possible and quality checked by the clinicians within the team. The presence of clinicians within the research team also ensure that the patient is the priority even with data storage and collection.
	**Theme 2: Visualizations and Diagnostic Tools** Tools like saliency maps, LIME, SHAP, and attention visualization have been discussed to provide insights into why AI models make specific decisions. *Supporting Code:* “By visualizing how models interpret inputs, we make AI decisions clearer and more understandable to end users.”	**Theme 2: Complexity and lack of understanding** This may prevent the general public from engaging fully with AI tools. *Supporting Code:* “People think AI is too complicated to understand, so I think they may feel uncomfortable using it.” **Our response:** We have incorporated a section which briefly outlines some of the technical information in more accessible terms. While this UI is designed to be used primarily with clinicians the principle also extends to patients being shown the UI outputs and hopes to enhance Ai literacy for patients and clinicians alike
	**Theme 3: Techniques for Bias Detection and Reduction** Computer scientists have also suggested ways of identifying and reducing biases in AI models, particularly in sensitive areas like healthcare. Techniques such as fairness constraints and debiasing were examples. *Supporting Code:* “We may incorporate fairness constraints into the training process to mitigate biases against underrepresented groups.”	
	**Theme 4: Creating Diverse Datasets** Recognizing that biases often stem from the data itself, there has been a push toward creating and curating more diverse datasets that better reflect the population. *Supporting Code:* “We may need to think of other datasets to ensure AI systems perform fairly across all demographics.”	

### User interface

To generate a User Interface that could be implemented clinically, we compartmentalised user interactions into three main areas using the R Shiny platform. No significant prior training is needed to allow users to generate an output, with walk-through tutorials built into the main interface and continued across each page of the tool. Users are simply required to select their preferred pre-loaded model and the clinical input data.

**Fig. 6:**
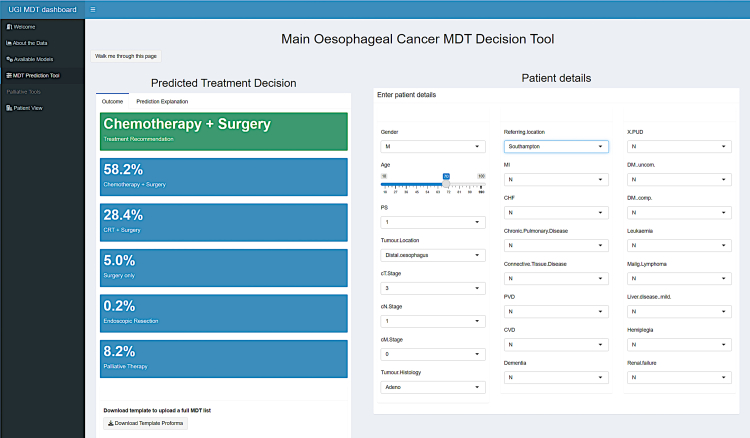
Primary Model interface and input screen.

Patient variables are inputted for the primary treatment model in the first instance where an instant recommendation is then provided along with predicted probabilities for all potential outcomes to illustrate how confident the final recommendation is (Fig. 6). LIME explanations are also given for the final prediction.

**Fig. 7:**
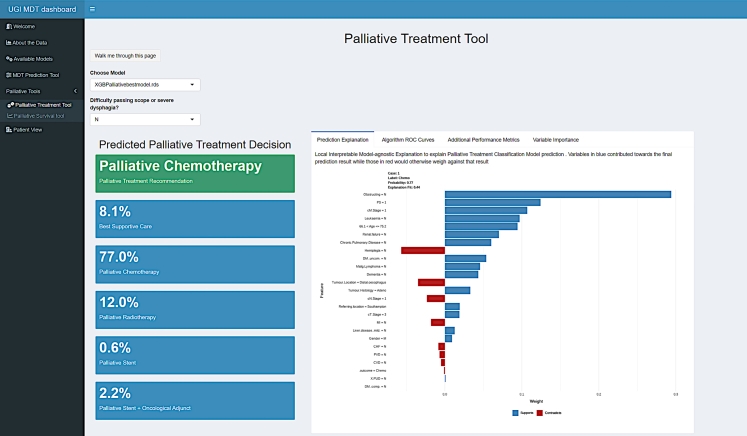
Palliative model recommendation and associated LIME explanation.

If the outcome is “Palliative”, the UI automatically carries the inputs across to the palliative treatment classifier (Fig. 7). As with the primary model, a LIME explanation is available for the user along with performance metrics for whichever model was loaded (RF, MLR, or XGB).

**Fig. 8:**
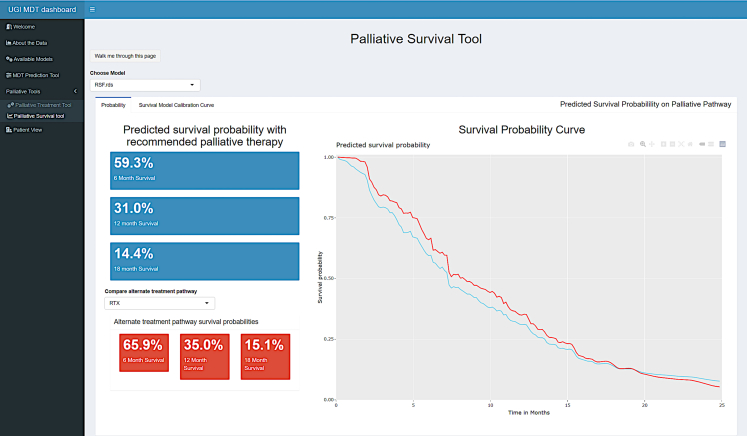
Palliative survival curves specific to recommended or selected treatment plans.

For palliative treatments, the associated predicted survival curve is then automatically generated along with an option to compare survival with an alternative pathway. In the example illustrated in Fig. 8, the original recommendation was for palliative chemotherapy which was then compared to palliative radiotherapy. The survival curves effectively personalise to the level of the treating hospital from which the training data was derived.

## Discussion

We present the first externally validated ML CDSS co-designed using RRI principles, capable of predicting OC MDT treatment decisions early within the cancer pathway. The sequential modelling approach quickly predicts a new patient's probable treatment plan which if palliative, is accurately prognosticated within the first 12 months post-diagnosis. All algorithms performed well; however, our results particularly favour MLR and XGB models, with mean AUCs above 0.87 for the primary classifier, and above 0.711 in the palliative classifier. The RSF model performed well within the first 12 months on calibration curve analysis and CRPS scores. Furthermore, the models have shown they generalise even when faced with differing cohort demographics. This suggests that for predictions at an instance-level, the ML models appear to handle perturbations in demographics at the feature-level. The use of a parallel RRI program ensured that the design of the AI CDSS has considered stakeholders and integrated their input. Early collaboration with a diverse skill-mix and open-format workshops have produced a CDSS with immediate clinical translational potential.

The primary treatment classifier models performed consistently well in discriminating palliative pathway patients across algorithms. This is primarily driven by the large palliative subgroup within the training process, a largely binary influence of cM staging and the additional input of high PS score patients. Across algorithms the models predict the curative pathways evenly however within the validation cohort (OUH) the CRT class demonstrates lower predictive performance. This is attributable to the relatively high use of CRT at UHS versus the OUH unit which favours neoadjuvant chemotherapy preferentially outside of squamous cell cancers. Until recently, chemotherapy and CRT have been equally acceptable options however early ESOPEC trial results now suggest chemotherapy may be the long-term front runner in these non-SCC cases.^28^ Where misclassifications occur this discordance between units favouring chemotherapy versus chemoradiotherapy is likely to be the main source. It is also important to recognise the differential in performance between the full primary classifier, which is trained off the full cohort, versus the palliative models which are only trainable on the palliative sub-group comprising approximately 50% of the training cohort. Classes such as palliative radiotherapy and best supportive care are innately harder to predict early on (radiotherapy is most commonly utilised for symptom control (dysphagia, pain, bleeding)) while chemotherapy is most prevalent as it provides disease control. BSC is determined on a combination of disease stage, physiological reserve and most importantly, patient wishes (the latter typically only determined after the MDT meeting when patients are seen in clinic).

The findings of this study continue to support the role of ML in oncological MDT decision-making, even early within the care pathway. The preserved performance on external validation indicates that overfitting remains modest. The optimal use of the palliative survival model was localised within the first 12 months post-diagnosis, in keeping with the expected survival for this cohort on current best therapy.^29^ As median survival was 6 months across the entire palliative cohort, and maximally 11 months with best palliative oncological therapy (chemotherapy) we have to acknowledge that predictions beyond the 1-year mark will not be reliable and return to best clinical judgement for those few who survive beyond this time point. As core management of OC is well established within national guidelines, ML lends itself to modelling the UK, decision-making framework, however this principle should in theory translate internationally as well.^22^^,^^30^ Where model performance drops between the cohorts this likely reflects idiosyncrasies specific to individual centres, an observation previously made in Denmark.^6^

There is a clear and urgent need to support MDT workflow. With 60% of new discussions likely to end in palliative treatment plans, rapidly predicting, and prioritising caseload is of clear clinical and financial benefit. When developed and implemented correctly they also have the capability to improve patient safety.^31^ Yet while AI-based CDSSs offer much to the healthcare sector, there remains a translational gap.^32^ This is multifactorial in nature but partly attributable to a sense of clinician superiority especially when handling nuance and uncertainty, or a lack of clinical validation.^33^ Furthermore, as the current boom in healthcare AI continues, the need for more responsible, co-designed and explainable AI (XAI) approaches are increasingly paramount.^19^ This study addresses this by establishing external validity of our clinical models combined with a co-designed user interface. Algorithms were chosen as either inherently interpretable (MLR) or amenable to both global and local XAI techniques.

The RRI program delineated potential challenges and barriers to the use of AI based CDSSs in clinical settings, including data bias, data governance, data drift, regulatory concerns over the role of the human-in-the-loop, and the foundations of legal and clinical responsibility.^34^^,^^35^ The new EU AI Act unsurprisingly classifies MAI into the “high-risk” category especially when the impact of new technologies may still be emerging far downstream of their first deployment (The “Collingridge” Dilemma).^36^^,^^37^

Our study employed one of the largest patient cohorts within the literature, providing robust internal and external validation of our models which span curative and palliative pathways. The algorithms are off-the-shelf libraries, ensuring that scalability of implementation is not reliant on high-performance computing clusters, something the current NHS digital infrastructure cannot offer evenly across the UK. The integrated RRI program was designed to act in the best interests of patients and clinicians. Including stakeholders early we have developed a highly functional CDSS which can be rationalised on a user-specific basis. The UI allows clinicians to counsel patients while insights derived from the program also allow development of future iterations of the CDSS. Consequently, the present study presents the first, cohesive, responsibly derived ML solution to assist OC MDT workflow needs which has not been provided previously within the literature. This is also the first study to externally validate OC treatment allocation models building on our previous efforts to integrate explainability within the process.^13^^,^^25^^,^^27^^,^^38^ Importantly, while previous studies have highlighted the utility of ML in other conditions,^11^^,^^12^^,^^18^^,^^39^ yet there often lacks a clear roadmap to guide the transition from technical demonstration to active clinical application. Here we have sought to provide a working tool that can be deployed online quickly and used by clinicians not specifically trained in ML.

One limitation was that the endoscopic management of early cancers had to be excluded from the validation analysis as we could not ensure a consistent selection criteria within the external cohort. Many cases are identified through Barrett's surveillance programs, and their care is not necessarily initiated by the MDT in the first instance making consistency of case presentation difficult. Additionally, we could not include novel molecular markers or immunotherapies within this generation of models as insufficient training data was available. Future iterations will support an expanding array of systemic treatments such as Chemotherapy ± anti HER2, anti-PD-1/PD-L1, Claudin 18.2, and immunotherapies for MMR-d/MSI-H tumours.^40^, ^41^, ^42^ As a newer cohort of patients emerge accruing data in these biomarkers, it is conceivable that these cases will be used to train a smaller model on just those features, the predicted probabilities of which may then be fed into a larger model leveraging the main cohort for whom those biomarkers may not have played a part in their treatment, reconciling the separate training datasets. Similarly, the recently reported ESOPEC trial may narrow down indications for NACRT, and future iterations will readily adapt to such trial outputs.^28^ While early curative cohort prognostication would be desirable (ideally prior to treatment initiation), the temporal effect of two separate major interventions (neoadjuvant therapy and subsequent surgery) make it extremely challenging in a single static model without post-operative inputs.^14^ It is also important to note that much of the data fed to MDTs may be recorded by non-clinical personnel or those of varying oncological experience especially in evaluating PS scores for patients. By way of example, while the OUH cohort may represent a fitter cohort, it is equally conceivable that less fit patients were either screened out pre-MDT in this unit or assigned lower PS scores erroneously. This also extends to data input as a whole, where prediction quality is inextricably linked to the quality of this input. While algorithms such as RF and XGB are capable of handling missing data, the user interface is designed to ensure all fields are completed. Fields set to a default and if left un-touched will still allow a prediction to be generated, however, it sits with the end-user to ensure that final inputs are correct else the prediction quality may be affected. Model fairness is not directly addressed within the scope of this study. Within the feature set only gender and age are protected characteristics, the latter of which we have previously investigated.^27^ However, it necessary to recognise that advanced age carries risk and clinician experience may easily be confused for bias in this context.^43^ Gender remains vulnerable to bias in OC too, which is historically a male-centred condition.^44^ Assessing model fairness regarding gender however requires assessing the equitability of the predicted outcomes which was beyond the scope of this study and evaluating long-term fairness of models will require more clarity in the definition of “equity” within OC treatment allocation. Finally, we have consciously chosen to map the current MDT versus an attempt to model the “best decision”. There remains no single, quantifiable metric currently agreed within OC to adequately encapsulate the myriad outcomes important to OC patients. Survival may not in every case be the most salient outcome measure, yet it is by far the most prolific in quantifying treatment “success” of oncological strategies. It is intended to be a springboard towards composite metrics which consider quality-of-life, complication rates or even resection margin status. Meanwhile, for this technology to translate to clinical use, we must first prove capable of mapping what “is” while the field attempts to agree upon what “should be”.

Future work in this field will look to integrate many of the novel markers discussed previously, as well as develop additional co-designed patient-only user interfaces. Broadening external validation to additional centres will further verify the results reported in this study. Trust must be established with patients, clinicians and regulators alike, and this study now sets the foundations for prospective trials within real-life scenarios to smooth the way towards clinical implementation. With the introduction of the EU AI Act, the regulatory landscape for medical AI continues to shift. Satisfying regulatory hurdles moving forward will almost certainly involve risk management, data governance, transparency, human oversight (and override mechanisms), post-market surveillance, quality management systems and CE marking among other considerations.^36^ Additional work will also be required to test such CDSSs in real-time clinical application. This will provide insight into if such a tool functions best when used within the MDT meetings or if is best utilised between meetings to triage discussions and “pre-screen” cases. Finally, an aspect lacking within the current literature is investigation into the decision-making thresholds for human agents faced with AI-based predictions in clinical settings across a range of machine confidence levels—at what confidence level is a clinician willing to accept and trust a prediction? And is this “line in the sand” equivalent for every use-case, patient or treatment? This will guide future Medical AI researchers when validating their model performances.

This is the first co-designed externally validated AI-derived CDSS targeted towards decision-making within the MDT cancer pathway for oesophageal cancer. It provides an integrated sequence of ML models which can reliably predict treatment allocation and palliative prognosis both locally and externally. The integration of an RRI program is intended to enhance user confidence that the CDSS considers individual and society risk as well as sources of potential bias within its design. Such technologies must contend with the standard challenges facing workflow integration within current digital healthcare infrastructures, as well as achieving clinician buy-in, especially where such models may adversely impact future clinician training. While future work includes prospective trials for real-world validation and regulatory approvals to address this, these models offer potential for a transformative impact on current MDT operations within the UK in OC and is both theoretically and technically transferrable to other cancer types and world regions.

## Contributors

**NT**–conceptualization, data curation, formal analysis, funding acquisition, investigation, methodology, project administration, resources, software, supervision, validation, visualization, writing—original draft, and writing—review & editing.

**MN**–data curation, formal analysis, methodology, visualization, writing—original draft, and writing—review & editing.

**MT**–conceptualization, formal analysis, methodology, software, validation, visualisation, and writing—review & editing.

**SAR**–data curation, formal analysis, methodology, software, validation, visualisation, and writing—review & editing.

**SLH**–methodology, project administration, and writing—review & editing.

**CP**–data curation, formal analysis, and writing–review & editing.

**ZSW**–funding acquisition, investigation, methodology, project administration, supervision, writing–original draft, and writing–review & editing.

**SR**–methodology, resources, supervision, and writing—review & editing.

**SM**–data curation, investigation, methodology, resources, validation, and writing—review & editing.

**RO**–data curation, investigation, methodology, resources, validation, and writing—review & editing.

**NM**–data curation, investigation, methodology, resources, validation, and writing—review & editing.

**TA**–investigation, methodology, validation, and writing—review & editing.

**ZB**–investigation, methodology, supervision, validation, and writing—review & editing.

**EVP**–conceptualization, investigation, methodology, resources, supervision, validation, and writing—review & editing.

**MM**–conceptualization, data curation, investigation, methodology, resources, validation, and writing—review & editing.

**TJU**–conceptualization, funding acquisition, investigation, methodology, project administration, resources, supervision, writing – original draft, and writing—review & editing.

**GV**–conceptualization, formal analysis, funding acquisition, investigation, methodology, project administration, software, supervision, validation, visualization, and writing—review & editing.

Authors NT and GV had access to and verified the underlying data.

All authors have read and approved the final version of the manuscript for publication.

## Data sharing statement

Data Availability: The data included within this study relates to sensitive intellectual property and cannot be shared freely at the present time.

Code Availability: The code used within this study includes sensitive intellectual property and cannot be shared freely at the present time.

## Declaration of interests

The Authors have no conflicts of interest to declare.
