# Supplementary material for: Oesophageal cancer multi-disciplinary tool: a co-designed, externally validated, machine learning tool for oesophageal cancer decision making
Source: eClinicalMedicine. 2025 Sep 30;89:103527. doi: 10.1016/j.eclinm.2025.103527 (PMC12513115; doi:10.1016/j.eclinm.2025.103527)
Supplement: Supplementary Materials [file mmc1.docx]

# Supplementary Materials

Contents

[Supplementary Materials 1](#_Toc207734837)

[Supplementary Methods 2](#_Toc207734838)

[Responsible Co-Design 2](#_Toc207734839)

[RRI prompts 2](#_Toc207734840)

[Co-design workshops 2](#_Toc207734841)

[Clinician Interviews 3](#_Toc207734842)

[Supplementary Results 7](#_Toc207734843)

[Supplementary Figures 7](#_Toc207734844)

[Supplementary Tables 11](#_Toc207734845)

[Final model hyperparameters 19](#_Toc207734846)

[Supplemental References 20](#_Toc207734847)

# Supplementary Methods

## Responsible Co-Design

The RRI program used in this study looked to approach the development of the ML-based CDSS in a number of separate but related strands. We utilised a semi-structured multidisciplinary workshop framework to discuss a series of RRI prompts from the RRI card deck devised by Horizon Digital Economy Hub (University of Nottingham). To obtain end-user insights we also conducted semi-structured interviews with MDT clinicians to understand the clinical variables they looked at during MDTs as well as how they perceived the use of AI-driven tools both in clinical medicine as a field as well as OC more specifically. They were also shown iterations of the evolving CDSS tool. The feedback and insights generated through this process was used to make changes and updates to the CDSS.

### RRI prompts

To ensure the CDSS development considered ethical, societal, and governance implications we utilised specially designed RRI card decks which prompts users to reflect, discuss and act on several aspects of RRI such as risk, bias, fairness, transparency ^7,8^. These also allow users to look ahead and anticipate potential consequences. This was used to inform the intended applications, target outcomes to predict and mechanisms through which to increase transparency and explainability of the CDSS at the point of use. The RRI card decks used within this program were designed by the University of Nottingham’s Horizon Digital Economy Hub (Supplemental Figure 4)^7,8^ . Each card is categorised under for one of the 4 principles of the AREA framework (Anticipate, Reflect, Engage, Act). A card will provide a specific prompt, as well as some example actions the research team can then use to action solutions. The cards cover a range of potential risks and issues including unintended consequences, sustainability, potential conflicts. Discussions were recorded and transcribed for later review and analysis.

### Co-design workshops

We conducted regular workshops attended by diverse members of the research group including digital health experts, XAI computer scientists, clinician scientists, Heartburn Cancer UK representatives, lay public and oesophageal cancer patients. These open-format workshops provided an arena for the research group to share thoughts on the evolution of the CDSS design while discussing validity of the models, clarity of the prediction explanations and the types of information patients may wish to know at the time of clinic review from their treating clinicians. RRI cards were again used here as stimulus prompts to consider new updates and evolutions of the CDSS between meetings and how these changes might mitigate or propagate risks associated with AI innovation. These workshops took place via teleconference over a series of months running in parallel with the UI development as well as validation of the ML models discussed within this study.

### Clinician Interviews

The development of the machine learning (ML) model and its associated user interface (UI) for our CDSS was also guided by semi-structed interviews with clinicians which were analysed by a thematic analysis (interview questions are included at the end of this section) ^9^. Key themes were identified, relating to their understanding, perceptions, and concerns about the use of AI in healthcare, specifically in the MDT of oesophageal cancer. The interviews were transcribed using Microsoft Teams software, and an iterative coding process was applied. Initially, open coding was used to identify significant statements and concepts. These codes were then grouped into broader themes through axial coding, which allowed for the identification of patterns and relationships within the data. The final themes were refined through selective coding, ensuring that they accurately represented the clinicians' perspectives and insights.

The interviews were structured into two key stages with the clinicians, focusing first on their general perceptions of AI in healthcare and later, specific feedback after interacting with the prototype tool. The semi-structured interviews engaged with six clinicians, including oncologists, radiologists, specialist nurses and surgeons specializing in oesophageal cancer. All participants are UK-based clinicians, and they have varied levels of experience in MDT ranging from 5 to 20 years. These participants were selected for their expertise and their roles in the multidisciplinary team of oesophageal cancer. Their involvement was crucial in ensuring the tool was aligned with the needs and workflows of end-users.

**Stage 1: Initial Interviews and Needs Assessment**

The first stage involved semi-structured interviews with the clinicians to explore their general understanding of AI and their perceptions of its potential role in clinical practice. The interviews aimed to identify clinicians' expectations, concerns, and perceived barriers to adopting AI tools in their workflow. Clinicians were asked about their familiarity with AI, their previous experiences (if any) with AI tools, and their views on the trustworthiness and reliability of AI in clinical decision-making. This stage provided critical insights into the clinicians' mental models regarding AI, which informed the initial design of the ML model and UI. Understanding the clinicians' baseline perceptions was essential for addressing any misconceptions and ensuring the tool was designed with their concerns in mind, particularly regarding transparency, explainability, and trust.

**Stage 2: Prototype Demonstration and Feedback**

In the second stage, after the initial design of the ML model and UI was completed, the clinicians were invited to participate in the second stage of interviews. During this stage, the clinicians were presented with a prototype of the tool, which included a preliminary version of the ML model integrated into a user interface. The clinicians were asked to interact with the tool and provide specific feedback on its functionality, usability, and clinical relevance. This feedback focused on the following areas:

- Usability: The ease of navigation within the UI, clarity of information presentation, and overall user experience.
- Functionality: The relevance and accuracy of the ML model’s predictions, as well as the usefulness of the tool in supporting clinical decision-making.
- Integration: How well the tool could be integrated into existing clinical workflows.
- Trust and Transparency: The clarity of the model’s explanations for its predictions and the degree to which clinicians felt they could trust and rely on the tool.

The insights gathered during these interviews were used to refine both the ML model and the UI. Specific changes were made to enhance the interpretability of the model’s predictions, improve the clarity of the user interface, and ensure that the tool met the clinicians’ practical needs in a clinical setting.

**Exploratory Clinician Interview materials (Questions)**

**ERGO number: 70375**

**IRAS: 319540**

**Clinician Interview primary goal:** To explore, clarify and consolidate the clinical variables expert clinicians intuitively utilise in determining clinical treatment pathways for the management of Oesophageal cancers (OC). We can subdivide these factors into clinical/ histopathological/ radiological and social variables.

**Clinician interview secondary goal:** Explore clinician perception, sentiment, and reservations for the inclusion of digital, Machine Learning (ML) and Artificial Intelligence (AI) - based clinical decision tools in health care settings, specifically the assessment and management of oesophageal cancer.

We aim to interview expert clinicians involved routinely in the management of oesophageal cancer patients. The inclusion criteria for our participants that they are current practicing consultant clinicians (Surgeons/ Gastroenterologists/ Oncologists) at the University Hospitals Southampton and experienced contributors to the Upper Gastrointestinal (UGI) Surgery Department multidisciplinary team (MDT) who are willing and agree to participate.

**Section 1: Each interview will discuss the following questions:**

Q1: What factors/clinical features do participants consider crucial to their decision-making? [Key decision-variables for downstream hybrid ML model]

Q1.1: What clinical features of the tumour complex would this include?

Q1.2: Are there any specific radiological factors that inform their decision-making process?

Q1.3: Do any specific histological features matter to the participant

Q1.4: Do participants consider any social or human factors routinely in choosing a treatment pathway?

Q1.4.1: If so, which? And why?

Q1.5: Of the factors discussed above, do participants attribute equal weighting to these or do they feel some factors are more important than others?

Q1.5.1: How would they rank such factors?

Q2: What do the participants understand by Machine Learning and Artificial Intelligence-based clinical decision tools? [pre-deployment perception]

Q3: What feelings/sentiments do participants possess towards these digital tools? [Current beliefs]

Q3.1 Do they currently use any automated tools outside UGI cancer clinics?

Q3.2What are their perception toward these tools?

Q4 What do they think AI could enable them to do in the clinic? [Perceived utility]

Q4.1 Do they currently feel they know enough to trust these tools in a healthcare setting?

Q5: What are the main barriers towards developing or possessing trust in such tools? [Barriers and expectations]

Q5.1 How advance do they think AI tools will be?

Q6: If such tools were available and scientifically validated what further safe-guards or measures would participants wish to have in place to feel willing to use such tools within their own practice? [Clinician confidence, motivation]

Q6.1 What motivates them to use AI tools in the clinic?

Q6.2 To what degree would clinicians be interested or willing to be part of the design and integration process?

# Supplementary Results

## Supplementary Figures


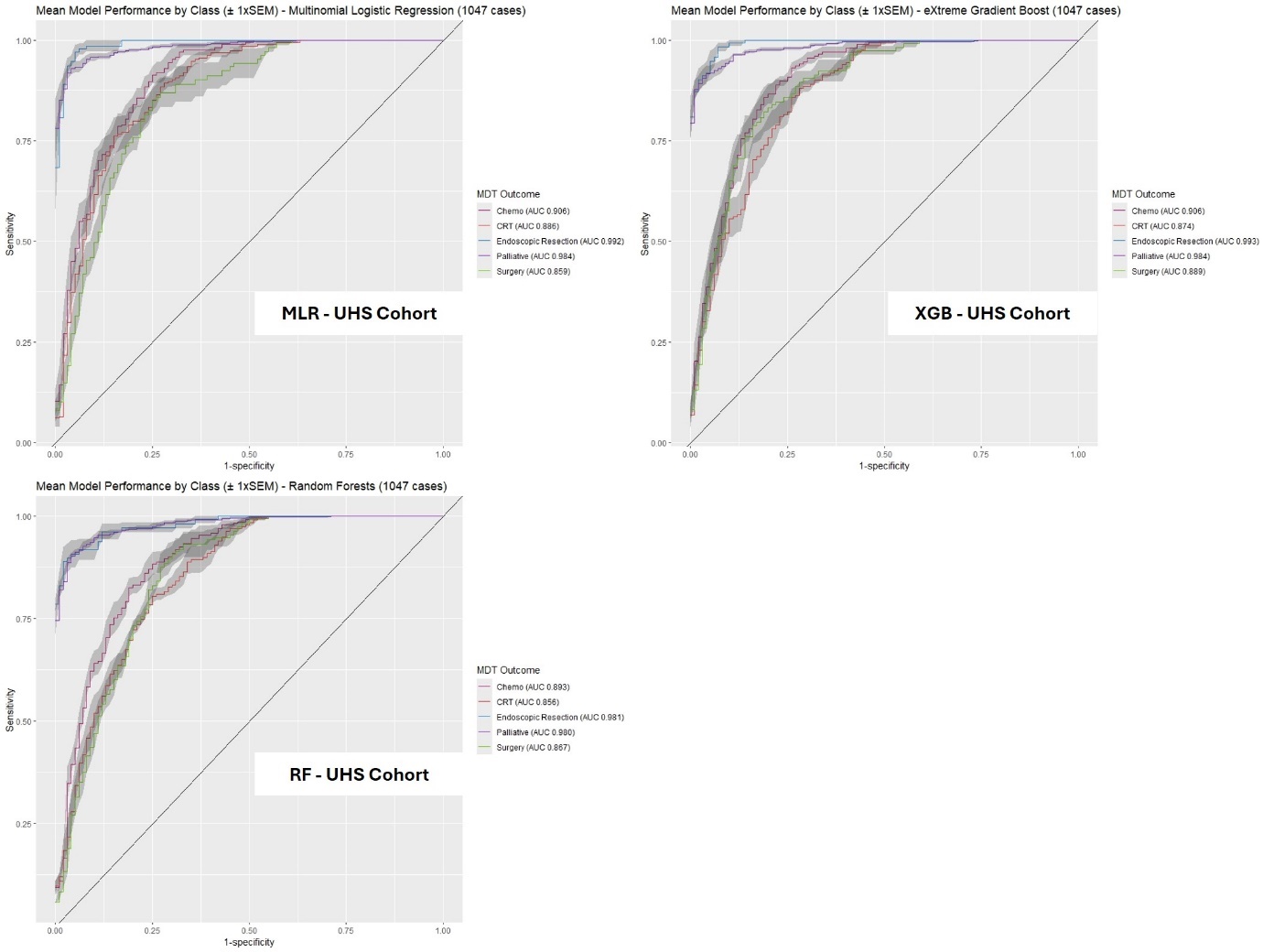


**Supplemental Figure 1 - Mean cross-validated ROC curves for each classifier algorithm (UHS cohort, 1047 cases) when model incorporates endoscopic resection class (N = 94). Shaded areas represent ±1x Standard error from the Mean.**


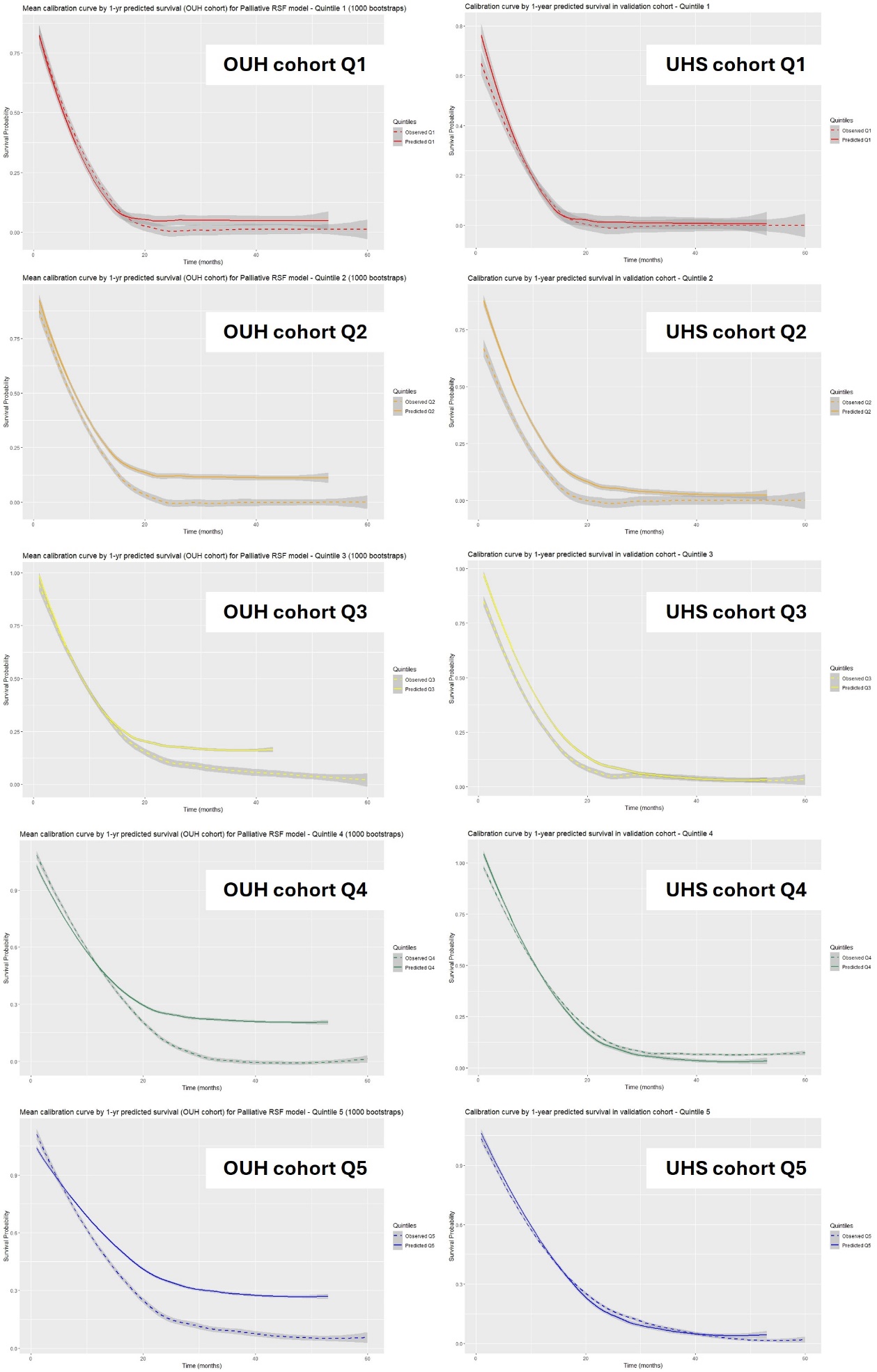


**Supplemental Figure 2 - Quintile Calibration curves for OUH survival model vs UHS validation cohort, plotted with standard error over 60 months with cases stratified by predicted 1-year survival probability (Quintile 1 = 0-20% (a), Quintile 2 = 20-40% (b), Quintile 3 = 40-60% (c), Quintile 4 = 60-80% (d), Quintile 5 = 80-100%)**


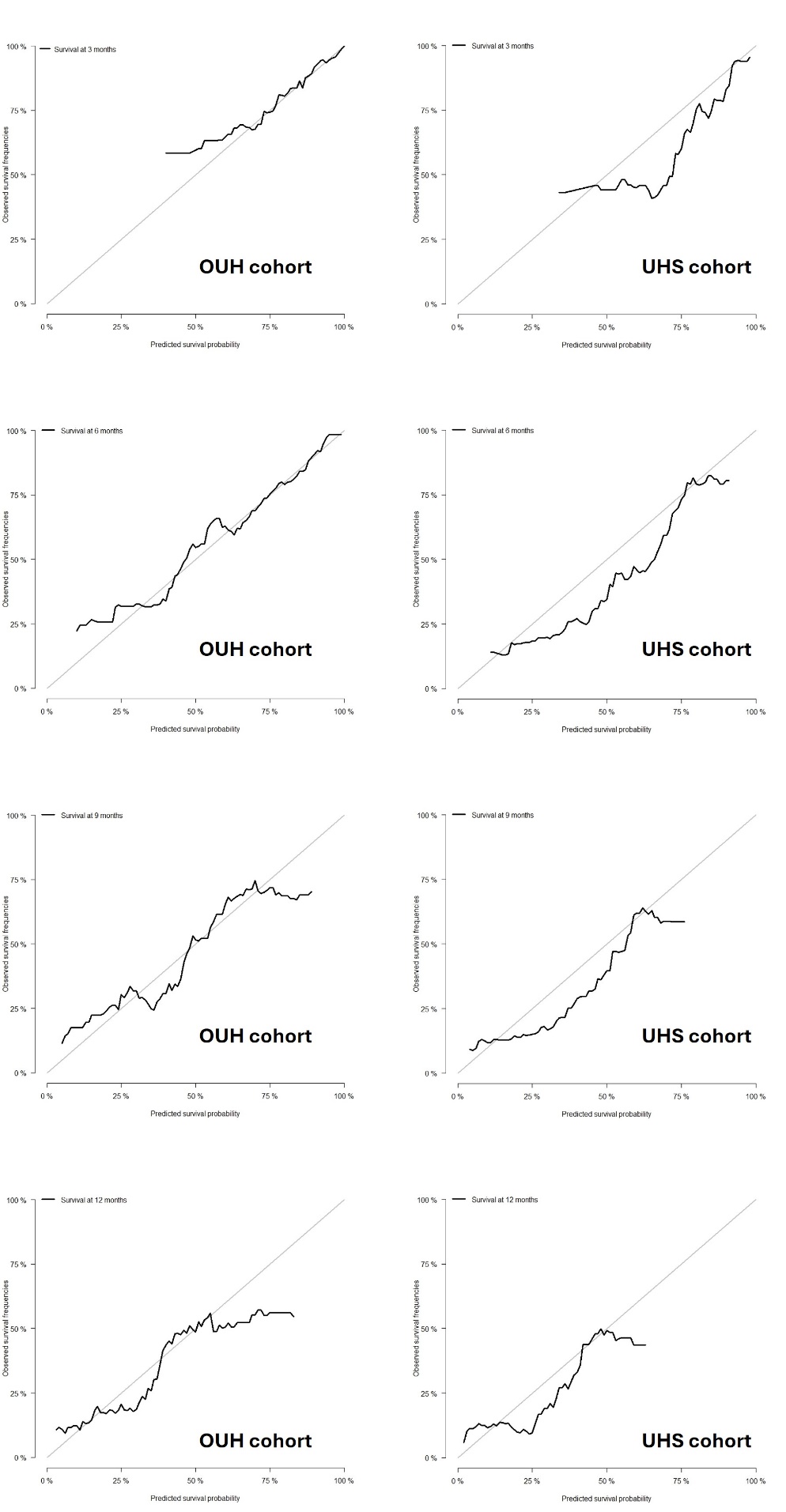


**Supplemental Figure 3 - Calibration plots for the OUH survival model (left) and UHS validation cohort (right) at 3,6,9 and 12 months post-diagnosis.**


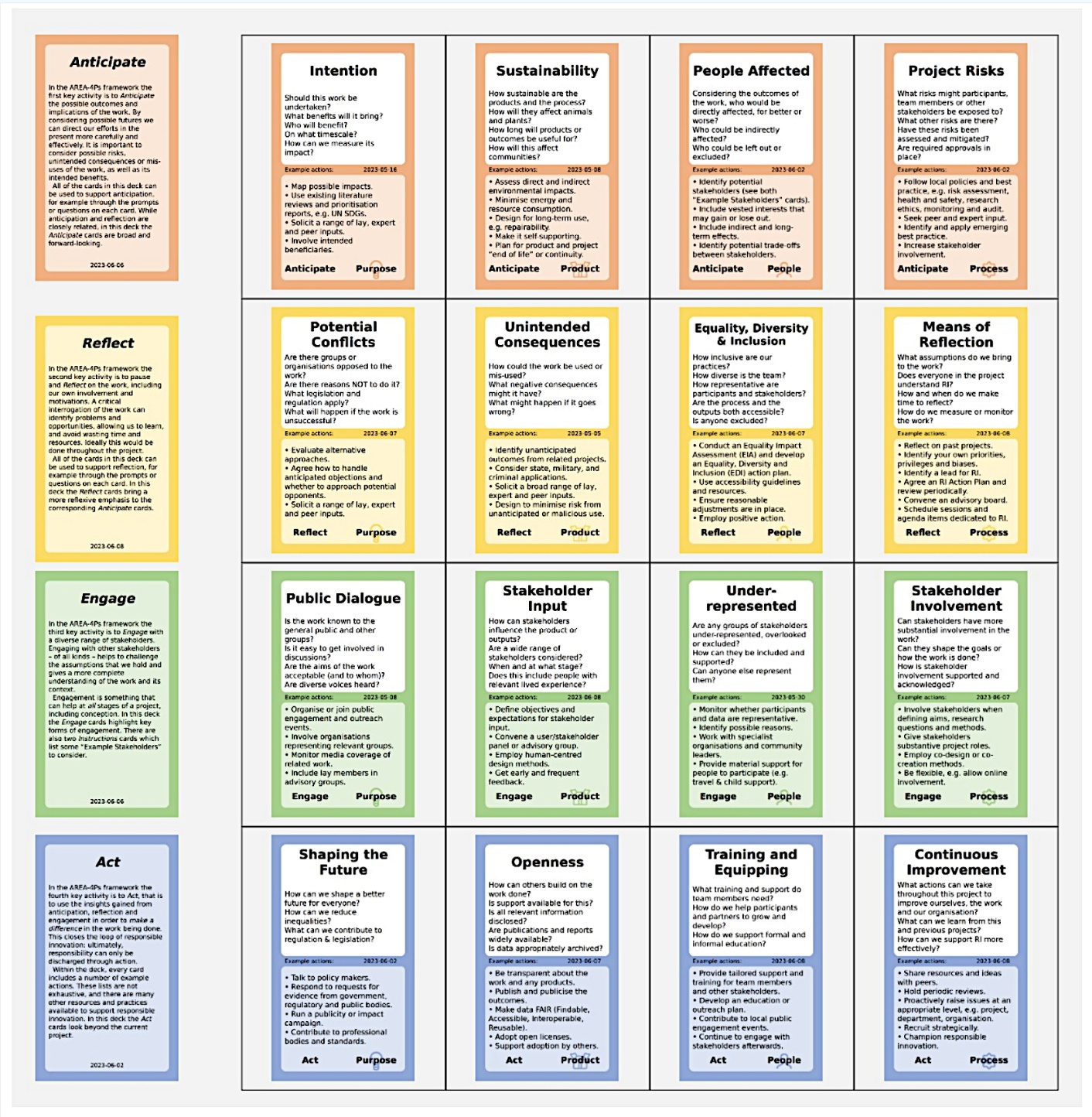


**Supplemental Figure 4 - RRI Card deck. Print friendly version available at http://doi.org/10.17639/nott.7353**

## Supplementary Tables

**Supplemental Table 1 - Patient demographics by model feature and primary model outcome classes.**

| **Pre-treatment variables** | **UHS “Chemo”**  **(N =210) (%)** | **OUH “Chemo”**  **(N =373) (%)** | **UHS “CRT”**  **(N =196) (%)** | **OUH “CRT”**  **(N =86) (%)** | **UHS “Surgery”**  **(N =108) (%)** | **OUH “Surgery”**  **(N = 44) (%)** | **UHS Palliative**  **(N =439) (%)** | **OUH Palliative**  **(N =475) (%)** | **UHS Total**  **(N = 953) (%)** | **Total**  **(N = 978) (%)** |
| --- | --- | --- | --- | --- | --- | --- | --- | --- | --- | --- |
| **Gender** | | | | | | | | | | |
| Male | 179 (85∙2%) | 303 (81∙2%) | 137 (69∙9%) | 52 (60∙5%) | 82 (75∙9%) | 34 (77∙3%) | 320 (72∙9%) | 355 (74∙7%) | 718 (75∙3%) | 744 (76∙1%) |
| Female | 313 (14∙8%) | 70 (18∙8%) | 59 (30∙1%) | 34 (39∙5%) | 26 (24∙1%) | 10 (22∙7% | 119 (27∙1%) | 120 (25∙3%) | 235 (24∙7%) | 234 (23∙9%) |
| **Median Age in years (Range)** | 65∙5 (21 – 81∙2) | 65∙0 (31∙0 – 80∙0) | 66∙6 (40∙0 – 81∙0) | 65∙0 (29∙0 – 77∙0) | 73∙4 (33∙7 – 83∙0) | 67∙5 (37∙0 – 83∙0) | 75∙2 (29∙8 – 96∙7) | 72∙0 (33∙0 – 96∙0) | 70∙0 (21∙0 – 96∙7) | 68 (29∙0 – 96∙0) |
| **Performance status** | | | | | | | | | | |
| 0 | 120 (57∙1%) | 350 (93∙8%) | 138 (70∙4%) | 83 (96∙5%) | 38 (35∙2%) | 39 (88∙6%) | 75 (17∙1%) | 240 (50∙5%) | 371 (38∙9%) | 712 (72∙8%) |
| 1 | 85 (40∙5%) | 22 (5∙9%) | 54 (27∙6%) | 3 (3∙5%) | 53∙7%) | 4 (9∙1%) | 132 (30∙1%) | 121 (25∙5%) | 329 (34∙5%) | 150 (15∙3%) |
| 2 | 5 (2∙4%) | 1 (0∙3%) | 3 (1∙5%) | 0 | 12 (11∙1%) | 1 (2∙3%) | 140 (31∙9%) | 69 (14∙5%) | 160 (16∙8%) | 71 (7∙3%) |
| 3 | 0 | 0 | 1 (0∙5%) | 0 | 0 | 0 | 87 (19∙8%) | 43 (9∙1%) | 88 (9∙2%) | 43 (4∙4%) |
| 4 | 0 | 0 | 0 | 0 | 0 | 0 | 5 (1∙1%) | 2 (0∙4%) | 5 (0∙5%) | 2 (0∙2%) |
| **cT stage** | | | | | | | | | | |
| 0 | 1 (0∙5%) | 0 | 0 | 0 | 2 (1∙9%) | 0 | 1 (0∙2%) | 0 | 4 (0∙4%) | 0 |
| Is | 0 | 0 | 0 | 0 | 3 (2∙8%) | 0 | 0 | 0 | 3 (0∙3%) | 0 |
| 1 | 0 | 1 (0∙3%) | 0 | 0 | 6 (5∙6%) | 1 (2∙3%) | 1 (0∙2%) | 0 | 7 (0∙7%) | 2 (0∙2%) |
| 1a | 0 | 0 | 0 | 0 | 1 (0∙9%) | 12 (27∙3%) | 0 | 1 (0∙2%) | 1 (0∙1%) | 13 (1∙3%) |
| 1b | 0 | 3 (0∙8%) | 0 | 0 | 1 (0∙9%) | 13 (29∙5%) | 0 | 1 (0∙2%) | 1 (0∙1%) | 17 (1∙7%) |
| 2 | 35 (16∙7%) | 107 (28∙7%) | 44 (22∙4%) | 19 (22∙1%) | 49 (45∙4%) | 11 (25∙0%) | 41 (9∙3%) | 59 (12∙4%) | 169 (17∙7%) | 196 (20∙0%) |
| 3 | 149 (71∙0%) | 211 (56∙6%) | 138 (70∙4%) | 53 (61∙6%) | 43 (39∙8%) | 4 (9∙1%) | 227 (51∙7%) | 235 (49∙5%) | 557 (58∙4%) | 503 (51∙4%) |
| 4 | 19 (9∙0%) | 1 (0∙3%) | 11 (5∙6%) | 0 | 2 (1∙9%) | 0 | 102 (23∙2%) | 6 (1∙3%) | 134 (14∙1%) | 7 (0∙7%) |
| 4a | 6 (2∙9%) | 47 (12∙6%) | 3 (1∙5%) | 13 (15∙1%) | 0 | 1 (2∙3%) | 28 (6∙4%) | 77 (16∙2%) | 37 (3∙9%) | 138 (14∙1%) |
| 4b | 0 | 1 (0∙3%) | 0 | 0 | 0 | 0 | 15 (3∙4%) | 71 (14∙9%) | 15 (1∙6%) | 72 (7∙4%) |
| X | 0 | 2 (0∙5%) | 0 | 1 (1∙2%) | 1 (0∙9%) | 2 (4∙5%) | 24 (5∙5%) | 25 (5∙3%) | 25 (2∙6%) | 30 (3∙1%) |
| **cN stage** | | | | | | | | | | |
| 0 | 41 (19∙5%) | 140 (37∙5%) | 64 (32∙7%) | 32 (37∙2%) | 59 (54∙6%) | 1 (2∙3%) | 90 (20∙5%) | 108 (22∙7%) | 254 (26∙7%) | 313 (32∙0%) |
| 1 | 138 (65∙7%) | 144 (38∙6%) | 112 (57∙1%) | 27 (31∙4%) | 42 (39∙9%) | 33 (75∙0%) | 145 (33∙0%) | 131 (27∙6%) | 437 (45∙9%) | 310 (31∙7%) |
| 2 | 31 (14∙8%) | 76 (20∙4%) | 19 (9∙7%) | 25 (29∙1%) | 6 (5∙6%) | 8 (18∙2%) | 127 (28∙9%) | 1551 (31∙8%) | 183 (19∙2%) | 253 (25∙9%) |
| 3 | 0 | 12 (3∙2%) | 3 (1∙5%) | 2 (2∙3%) | 1 (0∙9%) | 1 (2∙3%) | 59 (13∙4%) | 82 (17∙3%) | 61 (6∙4%) | 97 (9∙9%) |
| X | 0 | 1 (0∙3%) | 0 | 0 | 0 | 1 (2∙3%) | 18 (4∙1%) | 3 (0∙6%) | 18 (1∙9%) | 5 (0∙5%) |
| **cM stage** | | | | | | | | | | |
| 0 | 210 (100%) | 373 (100%) | 196 (100%) | 86 (100%) | 108 (100%) | 44 (100%) | 176 (40∙1%) | 209 (44∙0%) | 690 (72∙4%) | 712 (72∙8%) |
| 1 | 0 | 0 | 0 | 0 | 0 | 0 | 257 (58∙5%) | 263 (55∙4%) | 257 (27∙0%) | 263 (26∙9%) |
| X | 0 | 0 | 0 | 0 | 0 | 0 | 6 (1∙4%) | 3 (0∙6%) | 6 (0∙6%) | 3 (0∙3%) |
| **Tumour location** | | | | | | | | | | |
| Proximal Oesophagus | 0 | 1 (0∙3%) | 3 (1.5%) | 0 | 0 | 0 | 19 (4∙3%) | 19 (4∙0%) | 22 (2∙3%) | 20 (2∙0%) |
| Mid oesophagus | 6 (2∙9%) | 28 (7∙5%) | 22 (11∙2%) | 24 (27∙9%) | 10 (9∙3%) | 6 (13∙6%) | 64 (14∙6%) | 118 (24∙8%) | 102 (10∙7%) | 176 (18∙0%) |
| Distal Oesophagus | 120 (57∙1%) | 124 (33∙2%) | 148 (75∙5%) | 33 (38∙4%) | 67 (62∙0%) | 16 (36∙4%) | 252 (57∙4%) | 148 (31∙2%) | 570 (59∙8%) | 321 (32∙8%) |
| Siewert 1 | 24 (11∙4%) | 142 (38∙1%) | 8 (4∙1%) | 19 (22∙1%) | 4 (3∙7%) | 9 (20∙5%) | 20 (4∙6%) | 86 (18∙1%) | 56 (5∙9%) | 256 (26∙2%) |
| Siewert 2 | 39 (18∙6%) | 78 (20∙9%) | 10 (5∙1%) | 10 (11∙6%) | 19 (17∙6%) | 13 (29∙5%) | 56 (12∙8%) | 104 (21∙9%) | 124 (13∙0%) | 205 (21∙0%) |
| Siewert 3 | 23 (11∙0%) | 0 | 1 (0∙5%) | 0 | 5 (4∙6%) | 0 | 28 (6∙4%) | 0 | 57 (6∙0%) | 0 |
| Siewert undefined | 15 (7∙1%) | 0 | 4 (2∙0%) | 0 | 3 (2∙8%) | 0 | 0 | 0 | 22 (2∙3%) | 0 |
| **Tissue Histology** | | | | | | | | | | |
| Adenocarcinoma | 197 (93∙8%) | 343 (92∙0%) | 134 (68∙4%) | 45 (52∙3%) | 96 (88∙9%) | 40 (90∙9%) | 322 (73∙3%) | 352 (74∙1%) | 749 (78∙6%) | 780 (79∙8%) |
| Squamous Cell | 13 (6∙2%) | 30 (8∙0%) | 62 (31∙6%) | 41 (47∙7%) | 12 (11∙1%) | 4 (9∙1%) | 117 (26∙7%) | 123 (25∙9%) | 204 (21∙4%) | 198 (20∙2%) |
| Dysplasia | 0 | 0 | 0 | 0 | 0 | 0 | 0 | 0 | 0 | 0 |
| **Co-morbidities** | | | | | | | | | | |
| Chronic pulmonary disease (CPD) | 26 (12∙4%) | 63 (16∙9%) | 28 (14∙3%) | 15 (17∙4%) | 19 (17∙6%) | 10 (22∙7%) | 57 (13∙0%) | 91 (19∙2%) | 130 (13∙6%) | 179 (18∙3%) |
| Peripheral vascular disease (PVD) | 6 (2∙9%) | 6 (1∙6%) | 7 (3∙6%) | 2 (2∙3%) | 5 (4∙6%) | 0 | 25 (5∙7%) | 15 (3∙2%) | 43 (4∙5%) | 23 (2∙4%) |
| Cerebrovascular disease (CVD) | 8 (3∙8%) | 8 (2∙1%) | 6 (3∙1%) | 3 (3∙5%) | 8 (7∙4%) | 0 | 84 (19∙1%) | 33 (6∙9%) | 106 (11∙1%) | 44 (4∙5%) |
| Uncomplicated diabetes (DM uncomp) | 21 (10∙0%) | 57 (15∙3%) | 20 (10∙2%) | 11 (12∙8%) | 16 (14∙8%) | 10 (22∙7%) | 71 (16∙2%) | 77 (16∙2%) | 128 (13∙4%) | 155 (15∙8%) |
| Leukaemia | 0 | 1 (0∙3%) | 0 | 0 | 3 (2∙8%) | 0 | 7 (1∙6%) | 0 | 4 (0∙4%) | 1 (0∙1%) |
| Lymphoma | 1 (0∙5%) | 8 (2∙1%) | 2 (1∙0%) | 2 (2∙3%) | 3 (2∙8%) | 0 | 5 (1∙1%) | 3 (0∙6%) | 11 (1∙2%) | 13 (1∙3%) |
| Renal disease | 0 | 6 (1∙6%) | 1 (0∙5%) | 2 (2∙3%) | 3 (2∙8%) | 3 (6∙8%) | 35 (8∙0%) | 23 (4∙8%) | 39 (4∙1%) | 34 (3∙5%) |

**Supplemental Table 2 - Demographics for the Palliative training cohort (UHS) and validation cohort (OUH). Standardized Mean Differences (SMD) are provided for the two cohorts. An SMD of 0.2 is considered a small difference, 0.5 medium and 0.8 or more, a large difference**

| **Pre-treatment variables (Palliative)** | **UHS**  **(N =437) (%)** | **OUH**  **(N =475) (%)** | **SMD** |
| --- | --- | --- | --- |
| **Gender** | | | |
| Male | 318 (72∙8%) | 355 (74∙7%) | 0∙045 |
| Female | 119 (27∙2%) | 120 (25∙3%) |  |
| **Median Age in years (Range)** | 75∙2 (29∙8 – 96∙7) | 72∙0 (33∙0-96∙0) | 0∙172 |
| **Performance status** | | | |
| 0 | 74 (16∙9%) | 240 (50∙5%) | 0∙808 |
| 1 | 131 (30∙0%) | 121 (25∙5%) |  |
| 2 | 140 (32∙0%) | 69 (14∙5%) |  |
| 3 | 87 (19∙9%) | 43 (9∙1%) |  |
| 4 | 5 (1∙1%) | 2 (0∙4%) |  |
| **cT stage** | | | |
| 0 | 1 (0∙2%) | 0 | 0∙891 |
| Is | 0 | 0 |  |
| 1 | 1(0∙2%) | 0 |  |
| 1a | 0 | 1 (0∙2%) |  |
| 1b | 0 | 1 (0∙2%) |  |
| 2 | 40 (9∙2%) | 59 (12∙4%) |  |
| 3 | 227 (51∙9%) | 235 (49∙5%) |  |
| 4 | 102 (23∙3%) | 6 (1∙3%) |  |
| 4a | 28 (6∙4%) | 77 (16∙2%) |  |
| 4b | 14 (3∙2%) | 71 (14∙9%) |  |
| X | 24 (5∙5%) | 0 |  |
| **cN stage** | | | |
| 0 | 90 (20∙6%) | 108 (22∙7%) | 0∙274 |
| 1 | 143 (32∙7%) | 131 (27∙6%) |  |
| 2 | 127 (29∙1%) | 151 (31∙8%) |  |
| 3 | 59 (13∙5%) | 82 (17∙3%) |  |
| X | 18 (4∙1%) | 3 (0∙6%) |  |
| **cM stage** | | | |
| 0 | 176 (40∙3%) | 209 (44∙0%) | 0∙102 |
| 1 | 255 (58∙4%) | 263 (55∙4%) |  |
| X | 6 (1∙4%) | 3 (0∙6%) |  |
| **Tumour location** | | | |
| Proximal Oesophagus | 18 (4∙1%) | 19 (4∙0%) | 0∙799 |
| Mid oesophagus | 63 (14∙4%) | 118 (24∙8%) |  |
| Distal Oesophagus | 252 (57∙7%) | 148 (31∙6%) |  |
| Siewert 1 | 20 (4∙6%) | 86 (18∙1%) |  |
| Siewert 2 | 56 (12∙8%) | 104 (21∙9%) |  |
| Siewert 3 | 28 (6∙4%) | 0 |  |
| Siewert undefined | 0 | 0 |  |
| **Tissue Histology** | | | |
| Adenocarcinoma | 322 (73∙7%) | 352 (74∙1%) | 0∙010 |
| Squamous Cell | 115 (26∙3%) | 123 (25∙9%) |  |
| **Difficulty passing gastroscope and/or severe dysphagia** | | | |
| Yes | 222 (50∙8%) | 289 (60∙8%) | 0∙203 |
| **Co-morbidities** | | | |
| Chronic pulmonary disease (CPD) | 57 (13∙0%) | 91 (19∙1%) | 0∙167 |
| Peripheral vascular disease (PVD) | 25 (5∙7%) | 15 (3∙2%) | 0∙125 |
| Cerebrovascular disease (CVD) | 84 (19∙2%) | 33 (6∙9%) | 0∙370 |
| Uncomplicated diabetes (DM uncomp) | 71 (16∙2%) | 77 (16∙2%) | 0∙001 |
| Leukaemia | 1 (0∙2%) | 0 | 0∙068 |
| Lymphoma | 5 (1∙1%) | 3 (0∙6%) | 0∙055 |
| Renal disease | 34 (7∙8%) | 23 (4∙8%) | 0∙121 |

**Supplemental Table 3 - Comparison of cohort composition between UHS and OUH patients**

|  | **UHS (Training)** | **OUH (Validation)** |
| --- | --- | --- |
| **Gender** | Approximately 3:1 male: female distribution in both cohorts | |
| **Age** | UHS cohort slightly older (median Age 70 yrs) versus the Oxford cohort (median age 68) | |
| **Performance status** | PS scores were broadly distributed across PS0-2 in UHS cohort | PS scores were heavily weighted towards the PS0 in the OUH cohort, indicating a generally fitter population at presentation |
| **cT stage** | The majority of UHS and OUH cases presented with T3 disease with similar distributions across T2-4 however more UHS cases were coded as cT4(unspecified) while more OUH cases were specifically designated cT4a/b. | |
| **cN stage** | Similar distribution of N0-2 disease in both cohorts but a higher prevalence of N1 staging in UHS cohort. | |
| **cM stage** | Both cohorts presented with comparable distributions of cM0 vs cM1 disease | |
| **Tumour location** | The majority of UHS tumours were distal oesophageal | OUH tumours were evenly spread across both the distal oesophagus and the GOJ. |
| **Tumour Histology** | Distribution of histology was comparable in both cohorts with an approximately 80% of tumours OAC versus 20% OSCC | |
| **Co-morbidities** | Higher prevalence of cerebrovascular disease | Lower prevalence of Cerebrovascular disease. |

**Supplemental Table 4 - Primary classifier model performance over 1000 bootstraps**

| **AUCs** | Mean | Range | SD | 95% CI (one sample t-test) |
| --- | --- | --- | --- | --- |
| MLR | 0∙8665 | 0∙8176 - 0∙9149 | 0∙0145 | 0∙8656 - 0∙8674 |
| RF | 0∙8674 | 0∙8288 - 0∙9105 | 0∙0125 | 0∙8666 - 0∙8682 |
| XGB | 0∙8627 | 0∙8087 - 0∙9023 | 0∙0132 | 0∙8619 - 0∙8636 |

**Supplemental Table 5 - Statistical comparison of primary classifier model performance on Kruskal-Wallis analysis**

| **Overall p < 0.001** | MLR | RF |
| --- | --- | --- |
| RF | p = 0∙31 | - |
| XGB | p <0∙001 | p <0∙001 |

**Supplemental Table 6 - Mean classification AUCs for UHS model trained on 1047 cases with endoscopic resection class included (N = 94). Best performance for each class is highlighted in bold**

| UHS Model | Chemo | CRT | Surgery | Endo | Palliative | Mean |
| --- | --- | --- | --- | --- | --- | --- |
| MLR | **0∙906** | 0∙886 | 0∙859 | 0∙992 | **0**∙**984** | 0∙925±0∙060 |
| XGB | **0**∙**906** | 0∙874 | **0**∙**889** | **0**∙**993** | **0**∙**984** | **0**∙**929±0**∙**055** |
| RF | 0∙893 | 0∙856 | 0∙860 | 0∙981 | 0∙980 | 0∙914±0∙062 |

**Supplemental Table 7 - Palliative classifier model performance over 1000 bootstraps**

| **AUCs** | Mean | Range | SD | 95% CI (one sample t-test) |
| --- | --- | --- | --- | --- |
| MLR | 0∙7355 | 0∙6516 - 0∙8284 | 0∙0282 | 0∙7338 - 0∙7373 |
| RF | 0∙7808 | 0∙7159 - 0∙8402 | 0∙0197 | 0∙7780 - 0∙7821 |
| XGB | 0∙7989 | 0∙7339 - 0∙8806 | 0∙0202 | 0∙7976 - 0∙8001 |

**Supplemental Table 8 - Statistical comparison of palliative classifier model performance on Kruskal-Wallis analysis**

| **Overall p < 0.001** | MLR | RF |
| --- | --- | --- |
| RF | <0∙0001 | - |
| XGB | <0∙0001 | <0∙0001 |

**Supplemental Table 9 – Kaplan Meier survival estimator for the palliative UHS and OUH cohorts. Hazard ratios based on Cox’s Proportional Hazards provided with statistically significant differences in p values denoted by * if P <0.05, ** if P <0.01 & *** if P <0.001**

| UHS | | | | |  |
| --- | --- | --- | --- | --- | --- |
| **Treatment** | **N** | **Events** | **Median Survival (months)** | **95% CI** | **HR (95% CI)**  **(BSC as reference group)** |
| BSC | 56 | 56 | 2∙15 | 1∙3 – 3∙5 | - |
| Chemo | 148 | 134 | 11∙1 | 9∙7 – 12∙2 | 0∙27 (0∙20-0∙37)*** |
| RTX | 78 | 72 | 8∙4 | 7∙1 – 12∙9 | 0∙31 (0∙22-0∙44)*** |
| Stent | 113 | 113 | 3∙9 | 3∙1 – 4∙2 | 0∙77 (0∙56-1∙06) |
| Stent_Onc | 42 | 41 | 6∙0 | 4∙3 – 8∙5 | 0∙56 (0∙37-0∙84)** |
| OUH | | | | |  |
| **Treatment** | **N** | **Events** | **Median Survival (months)** | **95% CI** | **HR (95% CI)**  **(BSC as reference group)** |
| BSC | 34 | 25 | 5∙8 | 4∙4 – 15∙8 | - |
| Chemo | 147 | 122 | 11∙2 | 9∙9 – 12∙9 | 0∙73 (0∙47-1∙12) |
| RTX | 133 | 98 | 9∙7 | 8∙7 – 12∙0 | 0∙89 (0∙57-1∙39) |
| Stent | 86 | 76 | 4∙4 | 3∙7 – 5∙7 | 0∙56 (1∙12-2∙80)* |
| Stent_Onc | 75 | 68 | 6∙7 | 5∙7 – 8∙2 | 1∙46 (0∙92-2∙31) |

**Supplemental Table 10 - Mean classification AUCs for primary model using OUH as the training cohort and validating on UHS patients. Best performances by class are highlighted in bold both locally and in the validation cohort**

| Oxford model |  | “Chemo” | “CRT” | “Surgery” | “Palliative” | Mean (±SD) |
| --- | --- | --- | --- | --- | --- | --- |
| MLR | OUH | **0∙922** | **0**∙**834** | 0∙894 | 0∙972 | 0∙906±0∙058 |
|  | UHS Validation | 0∙853 | **0**∙**848** | 0∙782 | 0∙970 | 0∙863±0∙078 |
| XGB | OUH | 0∙917 | **0**∙**834** | **0**∙**924** | 0∙975 | **0**∙**913±0**∙**058** |
|  | UHS Validation | **0**∙**862** | 0∙829 | **0**∙**819** | **0**∙**975** | **0**∙**871±0**∙**072** |
| RF | OUH | 0∙910 | 0∙810 | 0∙851 | **0**∙**976** | 0∙887±0∙072 |
|  | UHS Validation | 0∙853 | 0∙780 | 0∙800 | **0**∙**975** | 0∙852±0∙086 |

**Supplemental Table 11 - Mean classification AUCS for palliative classifier model using OUH as the training cohort and validating on UHS patients. Best performances by class are highlighted in bold both locally and in the validation cohort**

| Oxford model |  | “Chemo” | “BSC” | “RTX” | “Stent” | “Stent_Onc” | Mean (±SD) |
| --- | --- | --- | --- | --- | --- | --- | --- |
| MLR | OUH | 0∙829 | **0**∙**803** | 0∙776 | **0**∙**707** | 0∙640 | 0∙751±0∙077 |
|  | UHS Validation | **0**∙**881** | 0∙659 | 0∙668 | 0∙712 | 0∙685 | 0∙721±0∙092 |
| XGB | OUH | **0**∙**831** | 0∙777 | **0**∙**787** | 0∙696 | **0**∙**661** | **0**∙**750±0**∙**070** |
|  | UHS Validation | 0∙872 | **0**∙**698** | 0∙722 | **0**∙**837** | **0**∙**731** | **0**∙**772±0**∙**077** |
| RF | OUH | 0∙818 | 0∙776 | 0∙779 | 0∙690 | 0∙603 | 0∙733±0∙087 |
|  | UHS Validation | 0∙844 | 0∙683 | **0**∙**725** | 0∙751 | 0∙646 | 0∙730±0∙075 |

**Supplemental Table 12 - Survival model performance metrics for OUH model and UHS validation cohorts**

| Metric | Cohort | Score | Reference | Interpretation |
| --- | --- | --- | --- | --- |
| Prediction error (1-Concordance) | OUH model | 0∙336±0∙021 | 0 = perfect concordance  1 = perfect non-concordance | Fair |
|  | UHS validation set | 0∙340 |  | Fair |
| CRPS (Integrated Brier Score/time) | OUH model | 0∙146±0∙017 | 0 = perfectly accurate model  1 = perfectly inaccurate model | Very Good |
|  | UHS validation set | 0∙101 |  | Very Good |

## Final model hyperparameters

| **Primary classifier algorithm** | **Hyperparameters** |
| --- | --- |
| MLR | Weight decay: 0 |
| RF | mtry: 6, ntree: 500 |
| XGB | nrounds: 50, max_depth: 2, eta: 0.3, gamma: 0, colsample_bytree: 0.6, min_child_weight: 1, subsample: 0.5 |

| **Palliative classifier algorithm** | **Hyperparameters** |
| --- | --- |
| MLR | Weight decay: 0 |
| RF | mtry: 6, ntree: 500 |
| XGB | nrounds: 50, max_depth: 1, eta: 0.3, gamma: 0, colsample_bytree: 0.6, min_child_weight: 1, subsample: 0.75 |

| **Palliative classifier algorithm** | **Hyperparameters** |
| --- | --- |
| MLR | Weight decay: 0 |
| RF | mtry: 6, ntree: 500 |
| XGB | nrounds: 50, max_depth: 1, eta: 0.3, gamma: 0, colsample_bytree: 0.6, min_child_weight: 1, subsample: 0.75 |

| **Palliative classifier algorithm** | **Hyperparameters** |
| --- | --- |
| RSF | Ntree: 1000, mtry: 17, nodesize: 2, nsplit: 10, splitrule: logrank |

# Supplemental References

1 National Institute for Health and Care Excellence. Oesophago-gastric cancer: assessment and management in adults NICE guideline. 2018 www.nice.org.uk/guidance/ng83.

2 Kwak SG, Kim JH. Central limit theorem: the cornerstone of modern statistics. *Korean J Anesthesiol* 2017; **70**: 144–56.

3 Rahman SA, Walker RC, Maynard N, *et al.* The AUGIS Survival Predictor: Prediction of Long-Term and Conditional Survival After Esophagectomy Using Random Survival Forests. *Ann Surg* 2023; **277**: 267–74.

4 Ishwaran H, Kogalur UB, Blackstone EH, Lauer MS. Random survival forests. *Annals of Applied Statistics* 2008; **2**: 841–60.

5 Harrell FE, Califf RM, Pryor DB, Lee KL, Rosati RA. Evaluating the yield of medical tests. *JAMA* 1982; **247**: 2543–6.

6 Brier GW. VERIFICATION OF FORECASTS EXPRESSED IN TERMS OF PROBABILITY. *Mon Weather Rev* 1950; **78**: 1–3.

7 Portillo V, Greenhalgh C, Craigon PJ, Ten Holter C. Responsible Research and Innovation (RRI) Prompts and Practice Cards: A Tool to Support Responsible Practice. In: ACM International Conference Proceeding Series. Association for Computing Machinery, 2023. DOI:10.1145/3597512.3599721.

8 Greenhalgh C, Craigon P, Portillo V, *et al.* Responsible Innovation (RI) Prompts and Practice Cards (version 3.1.1, November 2023). University of Nottingham Research Data Management Service. 2023; published online Nov 28. DOI:10.17639/nott.7353.

9 Braun V, Clarke V. Using thematic analysis in psychology. *Qual Res Psychol* 2006; **3**: 77–101.
